# Role of the CXCR4-LASP1 Axis in the Stabilization of Snail1 in Triple-Negative Breast Cancer

**DOI:** 10.3390/cancers12092372

**Published:** 2020-08-21

**Authors:** Boopathi Subramaniyan, Sangita Sridharan, Cory M. Howard, Augustus M.C. Tilley, Tupa Basuroy, Ivana de la Serna, Elke Butt, Dayanidhi Raman

**Affiliations:** 1 Department of Cancer Biology, University of Toledo Health Science Campus, Toledo, OH 43614, USA; boopathi.subramaniyan@utoledo.edu (B.S.); sangita.sridharan@utoledo.edu (S.S.); cory.howard@utoledo.edu (C.M.H.); augustus.tilley@utoledo.edu (A.M.C.T.); tbasuroy@mgh.harvard.edu (T.B.); ivana.delaserna@utoledo.edu (I.d.l.S.); 2 Cancer Center Division, Massachusetts General Hospital, Harvard Medical School, 149 Thirteenth Street, 7th Floor, Charlestown, MA 02129, USA; 3 Institute for Experimental Biomedicine II, University Clinic, 97070 Wuerzburg, Germany; butt_e@ukw.de

**Keywords:** CXCR4, LASP1, Akt, Snail1 stability, A20, GSK-3β

## Abstract

The CXCL12-CXCR4 axis plays a vital role in many steps of breast cancer metastasis, but the molecular mechanisms have not been fully elucidated. We previously reported that activation of CXCR4 by CXCL12 promotes the nuclear localization of LASP1 (LIM and SH3 protein 1). The nuclear LASP1 then interacts with Snail1 in triple-negative breast cancer (TNBC) cell lines. In this study, we report that the nuclear accumulation and retention of Snail1 was dependent on an increase in nuclear LASP1 levels driven by active CXCR4. The CXCR4-LASP1 axis may directly regulate the stabilization of nuclear Snail1, by upregulating nuclear levels of pS473-Akt, pS9-GSK-3β, A20, and LSD1. Furthermore, the activation of CXCR4 induced association of LASP1 with Snail1, A20, GSK-3β, and LSD1 endogenously. Thus, nuclear LASP1 may also regulate protein-protein interactions that facilitate the stability of Snail1. Genetic ablation of LASP1 resulted in the mislocalization of nuclear Snail1, loss of the ability of TNBC cells to invade Matrigel and a dysregulated expression of both epithelial and mesenchymal markers, including an increased expression of ALDH1A1, a marker for epithelial breast cancer stem-like cells. Our findings reveal a novel role for the CXCR4-LASP1 axis in facilitating the stability of nuclear localized Snail1.

## 1. Introduction

Breast cancer (BC) is a heterogeneous disease, and is the second leading cause of death in women among the cancer mortalities. Based on the new statistics from 2019, 3 out of 10 women will develop BC in their lifetime, and 1 in 7 will succumb to BC [1]. Mortality in BC patients is mainly due to metastasis to the lungs, bone, and brain. Triple-negative breast cancer (TNBC) is a highly aggressive form of BC, with an increased risk of relapse, drug resistance, and metastases. The durable or pathological complete response (pCR) is low in TNBC patients [2,3,4,5]. There is mounting evidence to suggest that the CXCL12-CXCR4 axis plays an important role in triple-negative breast cancer (TNBC) progression and metastasis [6,7,8,9,10,11]. Previously, we reported that the N-terminal LIM domain of LIM and SH3 protein 1 (LASP1) directly binds to CXCR4 through its C-terminal tail [12,13]. LASP1 is an adaptor protein, that is highly expressed in various cancer types, and is involved in cell migration when activated by CXCL12 [14]. Nuclear LASP1 expression is high in advanced and aggressive stages of breast cancer, and is a predictor of poor patient outcome [13,14,15]. Interestingly, CXCR4-mediated nuclear import of LASP1 could be blocked by antagonizing the activity of CXCR4, with its allosteric inhibitor AMD-3100 [13]. Furthermore, the stable silencing of LASP1 inhibited breast cancer cell invasion through Matrigel; altered the gene expression of cell junction and extracellular matrix proteins [14]. 

The epithelial mesenchymal transition (EMT) pathway plays a crucial role in embryonic development, tumor invasion, and metastasis [16,17]. During the EMT process, epithelial cells begin to express high levels of transcription factors, such as Snail1, Slug, Twist, and zinc finger E-box binding homeobox 1 and 2 (ZEB1 and ZEB2). This transcriptional network facilitates a phenotype in which cells reduce their intercellular adhesion and gain the ability to migrate and invade through the extracellular matrix [17,18,19]. A hallmark of EMT is the functional loss of E-cadherin, zona occludens-1 (ZO-1) and the concurrent upregulation of the mesenchymal markers, such as N-cadherin, vimentin and fibronectin.

Snail1, a zinc finger transcription factor, was originally identified as an E-cadherin repressor in *Drosophila melanogaster*, where it is mainly involved in controlling cell movement during mesoderm formation and neural crest delamination [20,21]. An elevated expression of Snail1 is commonly observed in breast cancer [19,22,23,24,25]. Moreover, Snail1 promotes EMT, and its increased expression level is highly associated with cancer stemness, relapse, chemoresistance and metastasis [26,27,28,29,30,31,32,33,34,35]. Snail1 epigenetically regulates metabolism in TNBC cells through the repression of transcription of fructose-1, 6-bisphosphatase (FBP1) gene. A subsequent reduction in the protein level of FBP1 promotes glycolysis in tumor cells [36]. Expression level of Snail1 is regulated by a variety of signaling pathways, including transforming growth factor-β (TGF-β), bone morphogenetic protein (BMP), epidermal growth factor (EGF), Notch, reactive oxygen species (ROS), hypoxia, and WNT signaling pathways [37,38,39]. However, Snail1 is a highly labile protein that undergoes ubiquitination by E3-ligases and subsequent cytosolic degradation [40]. Mechanistically, nuclear Snail1 is phosphorylated by glycogen synthase kinase-3β (GSK-3β), and is exported to the cytoplasm. In the cytosol, Snail1 becomes additionally phosphorylated and targeted for β-TRCP-dependent ubiquitination and degradation [41]. Similarly, the phosphorylation of nuclear Snail1 at Ser11 by protein kinase D1 (PDK1) primes it to undergo FBXO11-dependent ubiquitination in the nucleus and subsequent degradation in the cytosol [42,43]. 

Activation of CXCR4 triggers the phosphatidylinositol 3-kinase (PI3K) pathway, leading to the phosphorylation and activation of Akt [44]. Nuclear-localized phospho-Akt inhibits the degradation of Snail1 through inactivating phosphorylation of GSK-3β at Ser9 [42]. In addition, the nuclear stabilization of Snail1 is reported to depend on its monoubiquitination by A20 [45]. This post-translational modification on Snail1 is thought to reduce the affinity of GSK-3β for Snail1. This protects and stabilizes Snail1 from GSK-3β-mediated phosphorylation and subsequent degradation. Many repressive effects of Snail1 were mediated in concert with lysine specific demethylase 1 (LSD1) [46]. However, the role of LSD1 in the phosphorylation of Snail1 by GSK-3β is presently unclear. LSD1 may inhibit phosphorylation of Snail1 by physically shielding it from GSK-3β. In addition, the role of the CXCR4-LASP1 axis in contributing to nuclear stabilization of Snail1 remains unknown. In the current study, we demonstrate that both CXCR4-mediated signaling and nuclear localized LASP1 can facilitate the stabilization of nuclear Snail1. 

## 2. Results

### 2.1. Differential Expression of Snail1 and A20 in BC Cell Lines

**Figure 1:**
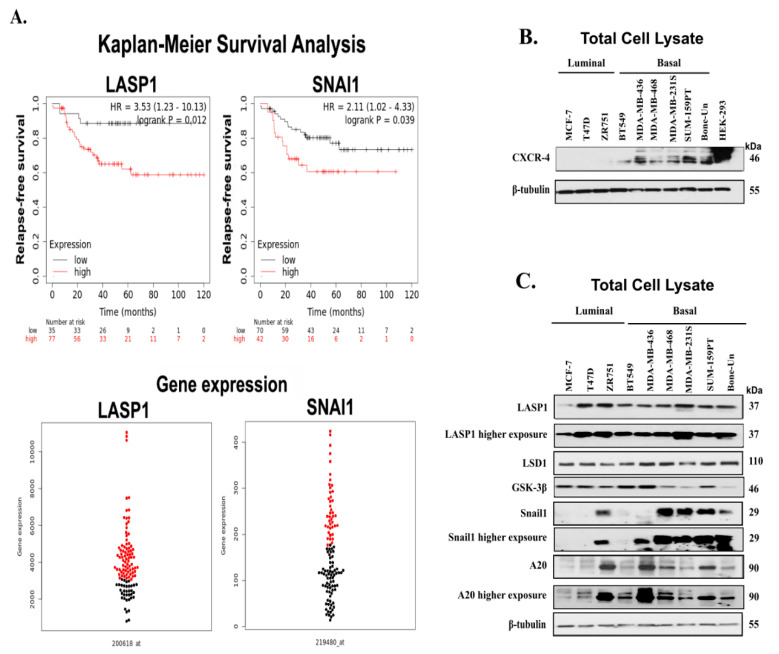
CXCR4 and Snail1 are expressed differentially in human breast cancer cell lines. (**A**) Top panel—Kaplan–Meier analysis of the relapse-free survival rate of basal-like, triple-negative breast cancer patients with grade 3 tumors, with a differential expression of *LASP1* and *SNAI1* (*n* = 112). Bottom panel—Beeswarm plot of differential gene expression profile of *LASP1* and *SNAI1*—red symbols—high expression; black symbols—low expression (**B**). Proteins in the total cell lysates were separated by 10% SDS-PAGE, and subjected to Western blot analysis for differential expression of CXCR4 by probing with anti-CXCR4 antibody in luminal and basal-like breast cancer cell lines. Moreover, β-tubulin served as the loading control (**C**) Proteins in the total cell lysates were separated by 10% SDS-PAGE and subjected to Western blot analysis for differential expression of proteins involved in Snail1 stabilization such as LASP1, LSD1, Snail1, and A20 in luminal and basal-like breast cancer cell lines. Furthermore, β-tubulin served as the loading control.

Before we correlate the expression of Snail1 and A20 in breast cancer cell lines, we performed Kaplan–Meier survival analysis to determine the probability of relapse-free survival when the expression of *LASP1* and *SNAI1* are high in human TNBC tumors. Interestingly, this study and others have found that the higher expression of *LASP1* and *SNAI1* was significantly correlated with lower relapse-free survival in TNBC patients with grade 3 tumors (Figure 1A, top panel *p* = 0.012 and a HR of 3.53 for *LASP1* and *p* = 0.039 and a HR of 2.11 for *SNAI1*) [47]. The differential distribution of gene expression profile of *LASP1* and *SNAI1* is depicted by the beeswarm plot [15] (Figure 1A bottom panel). Snail1 plays a major role with respect to chemoresistance, tumor initiation, and metastasis, but the protein is highly unstable. In order to determine the potential role of the CXCR4-LASP1 axis and other proteins such as A20 in the regulation of the Snail1 stability, we screened several human luminal and basal breast cancer cell lines, for the protein levels of CXCR4, LASP1, LSD1, GSK-3β, Snail1 and A20 by the immunoblot analysis of total cell lysates. We observed that CXCR4 is expressed in all of the basal-like BC (BLBC) cell lines correlating with their invasive potential. The protein was undetectable in luminal BC (LBC) cell lysates in our system. Ectopically expressed CXCR4 in human embryonic kidney-293 (HEK-293) cells served as positive control (Figure 1B). Next, we found that LASP1 is expressed uniformly high in both LBC and BLBC lines, with the exception of MCF7. LSD1 expression followed a similar profile to that of LASP1, with a tendency to higher levels in BLBC lines. Snail1 levels approximately mirrored the expression of CXCR4. A20 and GSK-3β are mostly expressed in all the cell lines, but we observed that the expression of GSK-3β was higher in MDA-MB-436 and SUM-159 PT. On the contrary, other BLBC cell lines showed lower expression of GSK-3β, further proving the importance of the MDA-Bone-Un cells (Figure 1C). GSK-3β is a well-known negative regulator for the Snail1 stability. Overall, the protein levels of CXCR4, Snail1, and A20 correlated the most, and only some cancer cell line-specific aberrations were observed. 

### 2.2. Activation of CXCR4 Signaling by CXCL12 Contributes to an Increased Stability of SNAIL1 through Modulation of Its Upstream Regulators 

We employed a bone-tropic metastatic variant of the TNBC cell line, MDA-MB-231 (which is denoted as ‘MDA-Bone-Un’ or simply Bone-Un) for all the experiments. Bone-Un cells were derived from the MDA-MB-231 cells obtained from murine bone lesions after intracardiac injection. These cells metastasize to the bones when orthotopically implanted into mammary fat pads [48,49]. 

**Figure 2:**
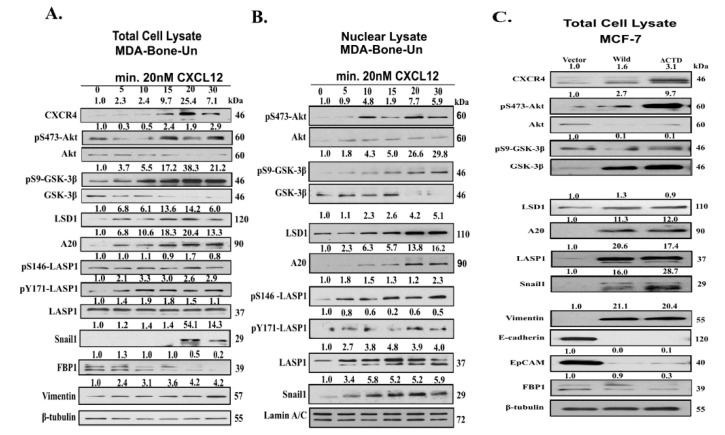
Nuclear Snail1 is stabilized upon activation of CXCR4 by CXCL12. (**A**) MDA-Bone-Un cells were stimulated with 20 nM CXCL12 for 0–30 min. The harvested (**A**) total protein lysates or (**B**) nuclear lysates were separated by 10% SDS-PAGE and subjected to Western blot analysis. The protein levels of Snail1 and proteins implicated in the Snail1 stabilization, such as AKT, GSK-3β, A20, LASP1, and LSD1 were analyzed and expressed as mean ± SD for 3 independent experiments (*n* = 3). Moreover, β-tubulin and Lamin A/C levels were employed as loading controls for total cell and nuclear lysate respectively. (**C**) MCF-7/Vector, MCF-7/CXCR4-WT, and MCF-7/ CXCR4-ΔCTD total protein lysates were subject to Western blotting. The protein levels of AKT, GSK-3β, LSD1, A20, LASP1, Snail1, and other EMT makers were measured and normalized to the levels of the β-tubulin as the loading control. The values were expressed as mean ± SD for 3 independent experiments (*n* = 3).

In order to elucidate the contributions of Akt, GSK-3β, LSD1, A20, and LASP1 to the stability of Snail1 when CXCR4 is activated, cells were incubated with 20 nM CXCL12, for various time points up to 30 min (Figure 2A). The level of CXCR4 peaked at 20 min, followed by a decline at 30 min, but still above the baseline level. Similar to this, the pS473-Akt level was elevated from 15–30 min above the baseline, with the levels normalized to total Akt. The level of pS9-GSK-3β increased steadily up to 20 min, followed by a minor decrease at 30 min with the levels normalized to total GSK-3β. There was only a marginal increase in the LSD1 after stimulation when compared to basal level until 30 min. The total levels of A20 increased in the first 20 min (20-fold increase), followed by a mild decline (13.3-fold) at 30 min. We then examined for the phosphorylation status of LASP1. Phosphorylation of LASP1 at S146 is known to induce nuclear translocation of the protein [50]. In addition, LASP1 contains a second phosphorylation site at Y171, which is reported to alter its cellular localization [51]. There was a marginal increase in the pS146-LASP1 levels at 20 min post stimulation, but the level of pY171-LASP1 increased up to 3.3-fold by 10 min, followed by a decrease to 2.9-fold at 30 min after stimulation. The phosphorylated forms of LASP1 were normalized to total LASP1 levels. 

Importantly, we examined the level of Snail1 following the stimulation of CXCR4 with 20 nM CXCL12. The levels of Snail1 in the total lysate dramatically increased by 20 min, which was followed by a decline at 30 min, but remained higher than basal level. This increase of Snail1 level correlated with a decrease in its transcriptional target fructose-1, 6-bisphosphatase (FBP1) and an increase in the mesenchymal cell marker vimentin. 

Next, we examined the levels of the aforementioned regulators of Snail1 stability in the nuclear fractions. We observed a CXCR4-stimulated increase in nuclear accumulation of pS473-Akt from 10–30 min (Figure 2B). The observed pattern of accumulation for pS473-Akt level mirrored that of pS9-GSK-3β. Furthermore, we observed an increase in nuclear accumulation of LSD1 and A20 as well. We observed an increase in nuclear localization of pS146-LASP1 similar to the expected lines of previous observations [2]. Interestingly, pY171-LASP1 exhibited a biphasic accumulation in the nucleus though the levels are not high. As reported by our previous findings [14], total LASP1 accumulated upon the stimulation of CXCR4 by CXCL12 (Figure 2B). We observed that the nuclear accumulation of LASP1 mirrored that of Snail1. 

### 2.3. Constitutive Activity of CXCR4 Increases Stability of Snail1 

In order to further assess the ability of CXCR4 to influence the stabilization of Snail1 levels, we employed a previously documented MCF7 series of cell lines. This includes vector-MCF7 (retroviral vector control) cells, WT-MCF7 (low ectopic expression of wild-type CXCR4) cells, and ∆CTD-MCF7 cells, which ectopically express a truncated CXCR4 that lack C-terminal with traditional phosphorylation sites [52]. In ∆CTD-MCF7 cells, the truncated CXCR4 is reported to demonstrate a gain-of function in signaling abilities. This was evident from a constitutively high ERK1/2 phosphorylation in the absence of CXCL12, and an observed increase in cell motility and mesenchymal markers. Importantly, ∆CTD-MCF7 cells were mesenchymal in morphology that was supported by the loss of classical epithelial markers, such as E-cadherin and the tight junction protein zona occludens-1 (ZO-1) [52]. Additionally, the ∆CTD-MCF7 cells demonstrated a high propensity for lung metastasis in a murine model [53]. 

Similar to our studies involving the stimulation of endogenous CXCR4, we observed an increase in the levels of pS473-Akt, A20, LASP1 and Snail1 in the constitutively active CXCR4 model. To support the notion that stabilized Snail1 was indeed active, we probed for EMT markers and observed corresponding reductions in the expression of epithelial markers, such as E-cadherin, epithelial cell adhesion molecule (EpCAM), and the metabolic enzyme FBP1. This was accompanied by an increase in the levels of the mesenchymal marker vimentin (Figure 2C).

Additionally, one dark band of the upregulated endogenous CXCR4 was observed to run a little higher, presumably due to phosphorylation. This was observed with the ectopic expression of constitutively active CXCR4 (Figure 2C). It is known that the activation of PI3K signaling pathway leads to translocation of phospho-Akt (pAkt) to the nucleus. Nuclear pAkt would then phosphorylate GSK-3β, which ultimately prevents the degradation of Snail1. We observed a 2.7 and 9.7-fold increase in pS473-AKT, with ectopic expression of WT and constitutively active CXCR4, respectively (Figure 2C). 

Interestingly, despite the upregulation of total GSK-3β in the presence of ectopically expressed WT or constitutively active CXCR4, the level of pS9-GSK-3β remained unchanged in this constitutive model. The levels of LSD1, A20 and total LASP1 were also elevated when WT or constitutively active CXCR4 are expressed in MCF-7 cells (Figure 2C).

Importantly, the levels of Snail1, as well as mesenchymal marker vimentin, were upregulated concomitant with a reduction in the expression of epithelial markers E-cadherin, epithelial cell adhesion molecule (EpCAM), and the metabolic enzyme FBP1 (Figure 2C).

### 2.4. LASP1 Associates with Snail1 and Other Proteins that Promote Snail1 Stability

**Figure 3:**
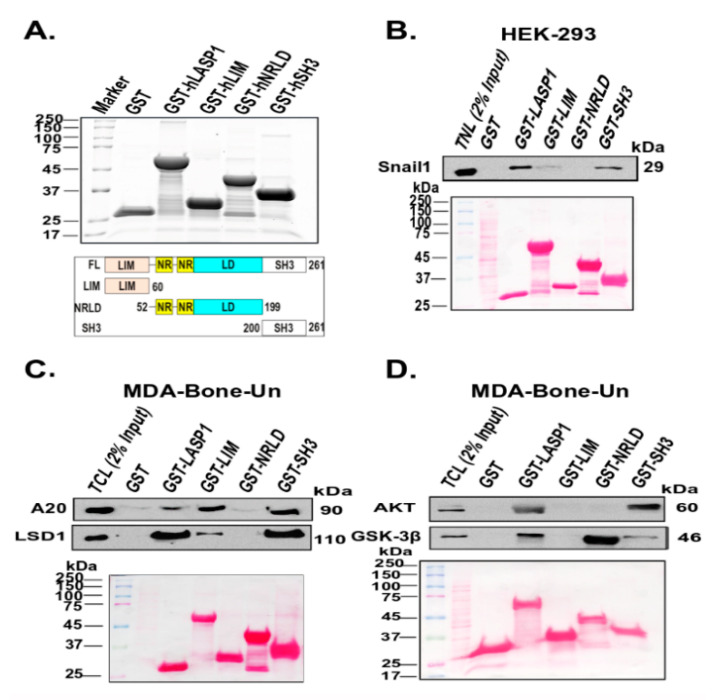
Domains of LASP1 differentially associate with Snail1 and its stabilizing proteins. (**A**) Top panel–Affinity purified full length LASP1 and its domains fused to GST were separated by 10% SDS-PAGE. Proteins were visualized by staining the gel with Imperial Blue Protein Stain; Bottom panel—A schematic depiction of the constructs of recombinant full length of LASP1 and its various domains that were bacterially expressed (**B**) Association of LASP1 and its domains with Snail1 upon stimulation of stably expressed CXCR4 by CXCL12. Notably, 1.5 nmol of each of the GST and LASP1 and its domains fused to GST were incubated with 200 µg of nuclear lysate derived from 293-CXCR4 cells that were transiently expressing Flag-Snail1-WT and stimulated with 20 nM CXCL12. The association of Flag-Snail1 with full length LASP1 and its domains were analyzed by 10% SDS-PAGE, followed by immunoblotting; *n* = 3 (**C**) and (**D**) Association of LASP1 and its domains with proteins that contribute to the stabilization of the Snail1. Nuclear lysates were prepared from MDA-Bone-Un cells that were serum-starved and stimulated with 20 nM CXCL12. 1.5 nmol of each of the GST and LASP1 and its domains fused to GST were incubated with 250 µg of nuclear lysate derived from CXCL12-stimulated MDA-Bone-Un cells. The association of endogenous A20, LSD1, AKT and GSK-3β with full length LASP1 and its domains were analyzed by 10% SDS-PAGE followed by immunoblotting; *n* = 3.

To further clarify the role of LASP1 in the stabilization of nuclear Snail1, we first investigated its association with known proteins which contribute to the stability of Snail1. This was accomplished through a glutathione S-transferase (GST) pulldown approach. We purified full length LASP1 and various domains of LASP1 fused to GST and examined for proteins that would associate from total lysates of MDA-Bone-Un cells (Figure 3A). Regions of LASP1 that associate with Snail1, A20, Akt, LSD1, and GSK-3β were identified. Previously, we reported that LASP1 associates with Snail1 endogenously in the nucleus only when TNBC cells were stimulated with CXCL12 [14]. Therefore, we employed HEK-293 cells with the ectopic stable and transient expression of CXCR4 and Flag-Snail1. Nuclear lysates were prepared following stimulation of CXCR4-HEK-293 cells with 20 nM CXCL12 and incubated with GST-full length LASP1 and its various domains. Both full length LASP1 and its SH3 domain robustly associated with nuclear Snail1. There was some association observed between the LIM domain of LASP1 and Snail1. The NRLD of LASP1 failed to associate with Snail1 under our conditions (Figure 3B). We then examined at the association of LASP1 with Snail1 regulators in MDA-Bone-Un lysates. The association of A20 mirrored that of Snail1, except for the protein more robustly associated with the LIM domain of LASP1. A similar binding pattern was detected for LSD1, i.e., association with full length LASP1 as well as with the LIM and SH3 domain, but not with the NRLD (Figure 3C). For Akt, association to the full length LASP1 and SH3 domain of LASP1 was observed. GSK-3β was the only protein that associated with the NRLD domain of LASP1 (Figure 3D).

### 2.5. Nuclear LASP1 Co-Immunoprecipitates with Snail1, A20, GSK-3β, and LSD1 

**Figure 4:**
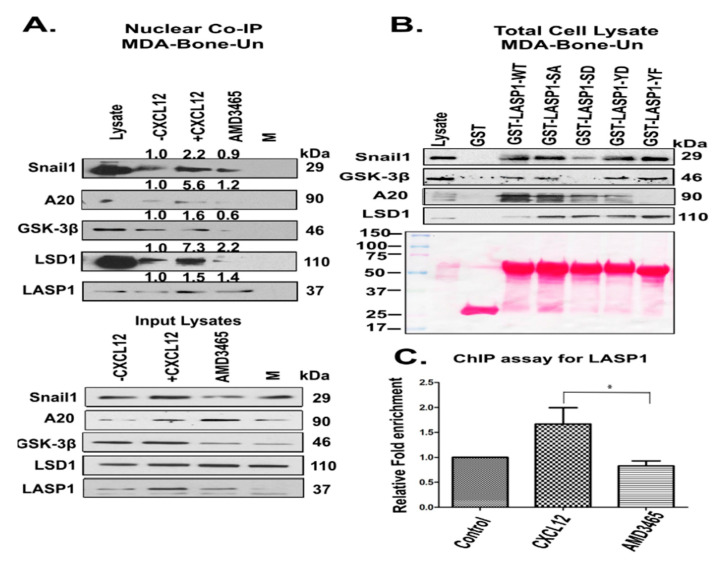
(**A**) Nuclear LASP1 co-immunoprecipitated with Snail1 and proteins that regulate the stability of Snail1 endogenously in response to CXCL12; (**A**) Co-immunoprecipitation of LASP1 with Snail1 and its regulators—Top panel: Serum-starved MDA-Bone-Un cells were stimulated with 20 nM CXCL12 for 20 min. In some conditions, Bone-Un cells were pre-incubated with the CXCR4 antagonist AMD-3465 for 30 min prior to the addition of the ligand CXCL12. LASP1 was immunoprecipitated with mouse anti-LASP1 (8C6 clone) antibodies from 250 µg of the nuclear extracts from MDA-Bone-Un cells and analyzed by 10% SDS-PAGE followed by immunoblotting for associated Snail1, A20, GSK-3β, and LSD1. ‘M’ represents the mock co-immunoprecipitation performed with isotype control IgG1 antibodies. Bottom panel: 15 µg of nuclear lysates were resolved by 10% SDS-PAGE, and immunoblotted for Snail1, A20, GSK-3β, LSD1 and LASP1 blot. (**B**) Differential association of phosphorylated forms of LASP1 to endogenous Snail1 and its regulators—1.5 nmol of each of the GST, LASP1-WT and its phosphomimetic (S146D and Y171D) and phosphonull (S146A and Y171A) mutants fused to GST were incubated with 250 µg of nuclear lysate derived from CXCL12-stimulated MDA-Bone-Un cells. The differential association of endogenous Snail1, GSK-3β, A20 and LSD1 with full length LASP1-WT and its mutants were analyzed by 10% SDS-PAGE, followed by immunoblotting; *n* = 3. (**C**) Occupancy of LASP1 at the E-cadherin promoter region—Serum-starved MDA-Bone-Un cells were stimulated with 20 nM CXCL12 for 20 min. In some conditions, Bone-Un cells were pre-incubated with the CXCR4 antagonist AMD-3465 for 30 min prior to the addition of the ligand CXCL12. The chromatin fragments were prepared and subjected to chromatin immunoprecipitation (ChIP) analysis. The data from quantitative, real-time PCR were statistically analyzed and shown as the Mean ± SD, *n* = 3 independent biological repeats; * *p* < 0.05.

To validate our findings from the GST pull-down approach, we performed a co-immunoprecipitation experiment, to examine the endogenous association of nuclear LASP1 with Snaill, A20, GSK-3β, and LSD1, when CXCR4 is activated. We indeed demonstrate that nuclear localized LASP1 associated with Snail1, A20, GSK-3β, and LSD1 endogenously in a CXCL12-dependent manner. Importantly, this association could be abrogated by treatment with AMD3465, supporting the specificity of CXCR4 (Figure 4A).

### 2.6. Differential Association of Phosphorylated Forms of LASP1 with Snail1, A20, GSK-3β, and LSD1

Next, we wanted to clarify the role of the phosphorylation status of LASP1 and its association with Snail1 and its regulators. We purified full length GST-LASP1 carrying various phosphomimetic and phosphonull substitutions at S146 and Y171. The phosphorylation status of LASP1 did not robustly regulate the LASP1-Snail1 association, although pS146D-LASP1 showed a lower affinity. Interestingly, this mutant did not associate with GSK-3β. On the contrary, A20 associated with all forms of LASP1, except for the Y171F mutant. Lastly, there appears to be no preferential association between the phosphomimetic and phosphonull mutations of LASP1 with LSD1, but the WT full length LASP1 did not associate very well (Figure 4B). 

### 2.7. Nuclear LASP1 Associates with the Promoter of E-cadherin in A CXCL12-Dependent Manner

Our previous [14] and current evidence had demonstrated a nuclear interaction between LASP1, Snail1, and its regulators. We next assessed the functional outcome of the nuclear localized LASP1 and its interaction with Snail1 and LSD1, by examining the occupancy of nuclear LASP1 at the E-cadherin promoter (a well-documented Snail1 and LSD1 target), using a chromatin immunoprecipitation (ChIP) assay. We observed a 2-fold enrichment of LASP1 at the E-cadherin promoter following stimulation with CXCL12. This enrichment was significantly abrogated by pre-treatment of MDA-Bone-Un cells with the CXCR4 antagonist AMD-3465 (Figure 4C).

### 2.8. Genetic Ablation of LASP1 Led to Loss of the Ability to Invade Matrigel

**Figure 5:**
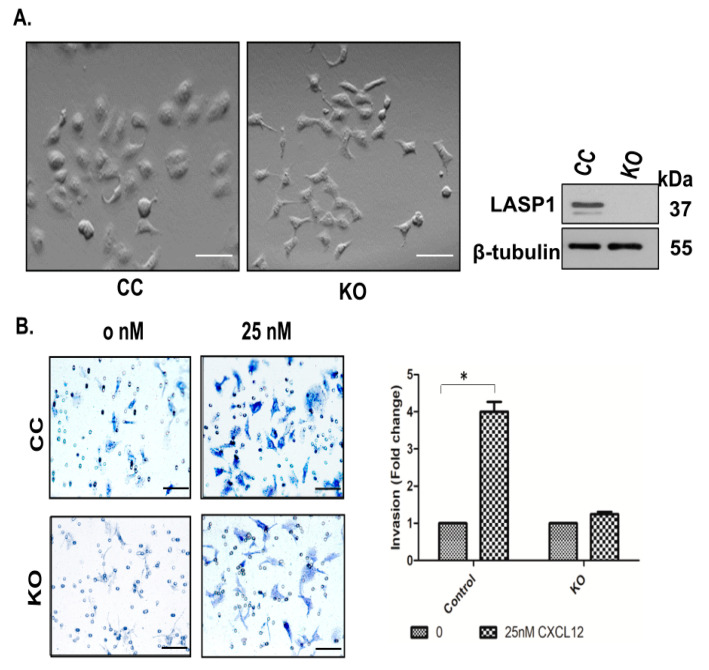

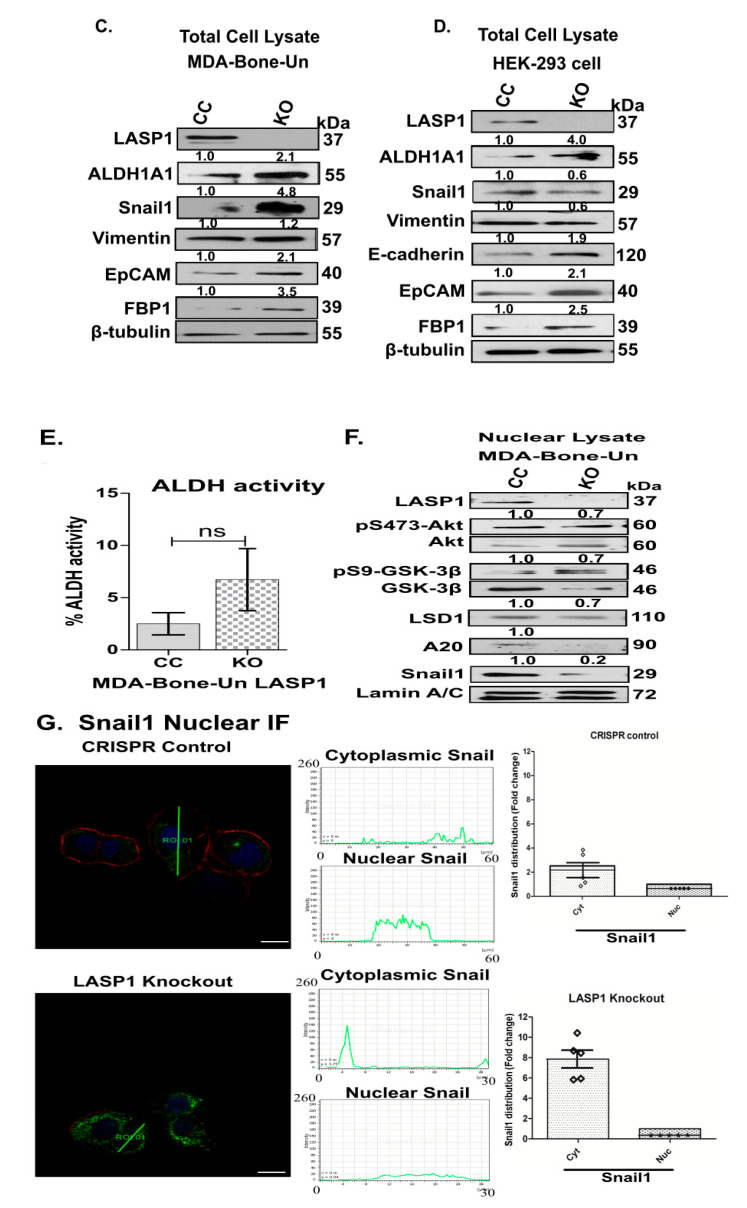
Genetic ablation of LASP1 impairs Matrigel invasion ability and alters the EMT program in TNBC cells. (**A**) LASP1 was genetically edited out by employing the CRISPR-Cas9 approach in MDA-Bone-Un cells. Left panel: The CC and LASP1-KO MDA-Bone-Un cells were plated in DMEM containing 10% serum. The light micrographs depict the morphology of CC and LASP1-KO cells. Right panel: the knockout of LASP1 was confirmed by Western blotting of total cell lysates of MDA-Bone-Un cells. Moreover, β-tubulin served as the loading control. Scale bar: 50 µM. (**B**) The CC and LASP1-KO MDA-Bone-Un cells (1 × 10^5^ cells) were seeded onto the upper chamber and allowed to invade through the Matrigel towards the lower chamber containing 25 nM CXCL12 as the chemoattractant overnight. The Matrigel, along with non-invaded cells in the upper chamber, were cleared off with a rubber policeman. The invaded cells that adhered to the bottom surface were fixed and stained with 0.1% crystal violet. The representative images for each condition were depicted (left panel). The cells in five randomly selected fields were counted and expressed as “Mean ± SD”, and the fold invasion is calculated and plotted (right panel); *n* = 3. Scale bar: 50 µM. (**C,D**) Altered epithelial and mesenchymal marker profile upon knockout of LASP1. Moreover, 25 µg of total cell lysate from CC and LASP1-KO in MDA-Bone-Un and HEK-293 cells were separated by 10% SDS-PAGE and the indicated epithelial and mesenchymal markers were analyzed by immunoblotting. β-tubulin served as the loading control. (**E**) Following LASP1 knockout in MDA-Bone-Un cells, ALDH activity was measured by FACS analysis. The given values represent mean ± SEM in 3 biological replicates (*n* = 3). (**F**) Altered profile of Snail1 and proteins involved in its stability upon knockout of LASP1. 25 µg of nuclear lysates from CC and LASP1-KO in MDA-Bone-Un cells were separated by 10% SDS-PAGE and analyzed by immunoblotting. Lamin A/C served as the loading control. (**G**) Mislocalization of Snail1 upon genetic ablation of LASP1 in MDA-Bone-Un cells. Cells seeded onto coverslips that were coated with collagen IV were serum-starved for 1 h and fixed. The subcellular localization of Snail1 (pseudo-colored green) was examined by immunofluorescent staining. DRAQ5 and Rhodamine-Phalloidin were employed to mark the nuclei and F-actin respectively. Confocal microscopic images were acquired and the distribution of the Snail1 was quantified using Image J software. Scale bar: 50 µM. Representative confocal micrographs of the cells that were subjected to quantification is depicted (left panel). The middle panel profiles the peak distribution of Snail1 in the representative images in the cytoplasm and nuclei of CC and LASP1-KO cells. The right panel displays the plots with fold change in the distribution of the Snail1 for CC and LASP1-KO cells. CC—CRISPR control, KO—LASP1 knock out. * *p* < 0.05.

To demonstrate the importance of LASP1 in the Snail1-EMT pathway, we knocked out LASP1 by employing a CRISPR-Cas9 approach in MDA-Bone-Un cells. Single cell colonies were isolated and showed no change in morphology (Figure 5A, left panel). Successful knockout was verified by immunoblotting (Figure 5A, right panel). Next, we investigated for the ability of the LASP1-KO MDA-Bone-Un cells to invade Matrigel. In CRISPR-control cells, incubation with 25 nM CXCL12 induced a 4-fold increase in the number of cells that invaded compared to unstimulated cells. On the contrary, we observed impairment in the invasion of LASP1-KO MDA-Bone-Un cells following CXCL12 stimulation (Figure 5B). 

### 2.9. Cytoplasmic Localization of Snail1 Following the Genetic Loss of LASP1

Finally, we examined for the level of Snail1 and the factors that affect its stability of Snail1 in LASP1-KO MDA-Bone-Un cells by immunoblotting total cell lysates. Surprisingly, the level of Snail1 protein was elevated by 4.8-fold when LASP1 was genetically ablated (Figure 5C). Importantly, we did not observe a similar elevation of vimentin, a protein normally reflecting the activity of Snail1. The epithelial markers EpCAM and FBP1 were elevated. In addition, we also observed an increase in the aldehyde dehydrogenase (ALDH) expression (Figure 5C) and activity (Figure 5E) following LASP1 knockout, which is indicative of a shift towards the epithelial phenotype [54], along with the retention of some mesenchymal markers. This further confirms that the cells exhibit a hybrid phenotype with both epithelial and mesenchymal characteristics. Moreover, the level of E-cadherin remained repressed. In contrast to LASP1-KO MDA-Bone-Un cells, the genetic loss of LASP1 in a model HEK-293 cell line showed the expected Snail1 downregulation by 0.6-fold. The levels of the epithelial markers, such as E-cadherin (1.9-fold), EpCAM (2.1-fold) and FBP1 (2.5-fold) were upregulated as expected. Concurrently, we also observed an increased ALDH1A1 expression (Figure 5D) and ALDH1A1 activity (Figure 5E). Under two-dimensional culture conditions, we analyzed for the levels of cytokeratin14 (CK14; a mesenchymal marker) and cytokeratin18 (CK18; an epithelial marker) upon genetic loss of LASP1. There was a marginal increase (1.5-fold) in CK18, but the level of CK14 was not altered (Figure S1). 

To further analyze the unexpected upregulation of Snail1 in MDA-Bone-Un cells after LASP1 knockout, we compared the levels of proteins that confer or affect the stability of Snail1 in nuclear lysates (Figure 5F). In the nuclear lysate, the levels of Akt, GSK-3β, LSD1 and A20 were downregulated upon genetic loss of LASP1. The level of pS473-Akt was reduced by 0.5-fold, and a marginal decrease in pS9-GSK-3β was noticed (Figure 5F). 

**Figure 6:**
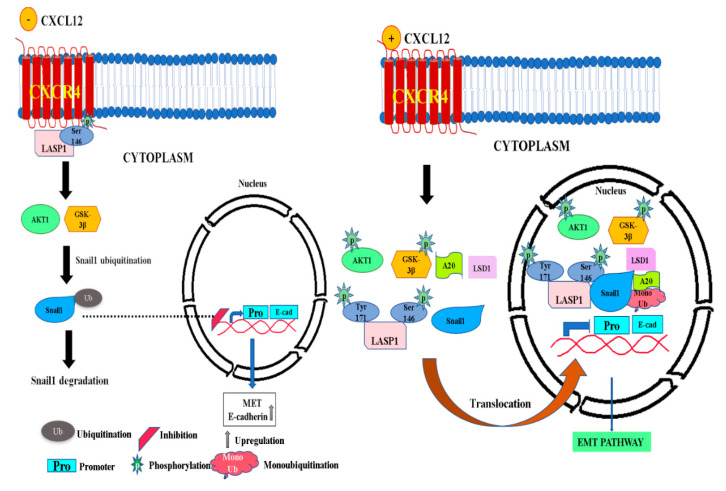
A working model illustrating the role of CXCR4-LASP1 axis in promoting the stabilization of nuclear Snail1. Left panel: A proposed model illustrating the cellular situation when CXCL12 is absent—In the absence of any activation of CXCR4 by CXCL12, the constitutively phosphorylated LASP1 at S146 (pS146-LASP1) is bound to inactive, unliganded CXCR4 at the plasma membrane. Phosphorylation of Snail1 by GSK-3β on specific residues results in cytosolic localization of Snail1. Cytoplasmic Snail1 undergoes additional phosphorylation by GSK-3β, which marks Snail1 for ubiquitination and subsequent proteasomal degradation. Gene expression of E-cadherin is not repressed by Snail1, though repression by other transcription factors such as ZEB1 is not ruled out. This happens in epithelial cancer cells. Right panel: Activation of CXCR4 by CXCL12 leads to the phosphorylation of LASP1 at Y171 and translocation of LASP1. Additionally, active CXCR4 induces phosphorylation/activation of Akt (pS473-AKT) through the PI3K pathway, which traverses to the nucleus and initiates the phosphorylation/inhibition of nuclear GSK-3β (pS9-GSK-3β). Concurrently, the CXCR4 signaling increases the nuclear levels of A20 and LSD1. Each of these proteins induces the stabilization of Snail1 in a variety of ways. A20 mediates monoubiquitination of Snail1 thus reducing its affinity for GSK-3β. Nuclear shuttled LASP1 and elevated LSD1 level may physically shield Snail1 and prevent access of GSK-3β to Snail1. The association of pLASP1 with Snail1, pAKT, A20 and LSD1 in the form of several complexes of differing compositions will protect and stabilize Snail1. The stabilized Snail1 represses the promoter region of E-cadherin. This happens in mesenchymal cancer cells. Snail1: Snail family transcriptional repressor; LASP1: LIM and SH3 protein 1; Akt: Protein kinase B; GSK-3β: Glycogen synthase kinase-3β; LSD1: lysine-specific histone demethylase 1; A20: TNF-α induced protein 3; E-cad: E-cadherin; Ub: Ubiquitination.

To corroborate this upregulation in the cytoplasmic and downregulation in the nuclear levels of Snail1, we examined the subcellular distribution of Snail1 upon the genetic loss of LASP1 by confocal microscopy (Figure 5G). Immunofluorescent staining of Snail1 in CRISPR-control and LASP1-KO MDA-Bone-Un cells revealed a pattern which matched the distribution observed in the biochemical fractionation experiments involving nuclear and cytosolic fractions. Figure 6 depicts a pictorial representation of the CXCR4-LASP1 axis contributing to Snail1 stability.

## 3. Discussion

In this study, we dissected the contributions of the CXCR4-LASP1 axis on the stability of Snail1 in TNBC cells. Snail1 is a key EMT-transcription factor that regulates cell migration, invasion, breast cancer stemness, and drug resistance in clinical TNBC cases of tumor relapse and therapy failure [30,55,56]. Therefore, the regulation of Snail1 is also critical for the control of primary tumor progression and for the regulation of many steps in metastasis [56]. In the present study, we observe upregulated expression levels of CXCR4, A20, and Snail1 in basal-like TNBC cells, suggesting that these proteins might be involved in the stabilization of Snail1. A20 is known to promote the stability of Snail1 through its monoubiquitination of Snail1 [45,57]. Nuclear pS473-Akt is an active serine kinase that stabilizes Snail1 through the inactivating phosphorylation of nuclear GSK-3β on S9. We observed that, in the presence of active CXCR4, the levels of both pS473-Akt and pS9-GSK-3β increased, indicating that signaling from CXCR4 might stabilize Snail1. The previous reports indicate that GSK-3β actively controls the localization of Snail1 through the phosphorylation of Snail1 protein which targets it for cytoplasmic degradation [41]. Thus, inactivation of GSK-3β enzymatic activity by CXCR4 signaling would have significant impact on Snail1 stability. We indeed observed an increase in the level of Snail1 around 20–30 min post stimulation with CXCL12. A similar observation was made in the constitutively active CXCR4 model in MCF7 cells. Recently, it has been shown that pS146-LASP1 can bind to CXCR4 at the plasma membrane and upon activation of CXCR4, LASP1 is released and traverses to the nucleus [13,51]. Interestingly, we made a novel observation that the phosphorylation of LASP1 on Y171 increases upon activation of CXCR4 in TNBC cells. In normal HEK cells, this phosphorylation is less dramatic [58], maybe due to the lower expression of tyrosine kinases in non-cancer cells. 

In all, the phosphorylation status of LASP1 may be context dependent. To investigate the importance of p-LASP1 in our system, we employed GST-LASP1 pulldown studies, with phosphomimetic substitutions demonstrating that LASP1, phosphorylated on either S146 [50] or Y171 [59], can indeed associate with Snail1 (though Snail1 associated with pS146-LASP1 with a reduced affinity). It is possible that the nuclear association of LASP1 with Snail1 can physically shield it from GSK-3β-mediated phosphorylation. Additionally, through its association with LSD1, LASP1 may form a LASP1-Snail1-LSD1 tripartite complex, which could additionally protect Snail1 from GSK-3β [46,60,61,62,63]. Eventually, nuclear LASP1 may also stabilize Snail1 by orchestrating its monoubiquitination via A20. Interestingly, the levels of A20 and LSD1 plummeted upon knockout of LASP1 in MDA-Bone-Un cells. We observed the association of AKT to the SH3 domain of LASP1, while another study found that the NRLD domain of LASP1 interacted with AKT [51]. This may be due to the stimulation of our TNBC cells, which led this differential association of the domains of LASP1 to AKT. In fact, LASP1 is nuclear in high grades of breast cancer. Nuclear localization of LASP1 and the 10-year survival rate are inversely correlated; i.e., poor patient outcome with the increasingly nuclear localized LASP1 [14,15]. In support of this, we observed that the high gene expression of LASP1 and SNAI1 in grade 3 tumors correlated with low probability of RFS in a cohort of 112 TNBC patients that we described in Figure 1A. The nuclear co-abundance of LASP1 and Snail1 in high grade tumors will most likely increase the propensity for local invasion and metastasis. 

To support the notion that the activity of Snail1 was indeed impaired in LASP1 KO cells, we examined the downstream EMT characteristics in these cells. Bone-Un LASP1 KO cells had an impaired ability to invade Matrigel. Importantly, the levels of epithelial markers and the transcriptional repressor targets for Snail1 such as EpCAM and the metabolic enzyme FBP1 increased [36]. Moreover, we observed an increase in ALDH-positive epithelial cancer stem-like cells. At the same time, the vimentin level remained the same, implicating a possible hybrid phenotype, having both epithelial and mesenchymal markers in TNBC cells [64,65]. Importantly, Snail1 was predominantly localized to the cytosol and depleted in the nucleus. 

We are also the first, to the best of our knowledge to provide the functionality of the LASP1-Snail1 interaction by demonstrating that LASP1 occupied the E-cadherin promoter in a CXCL12-dependent manner, probably as a co-factor together with Snail1 and LSD1, shown here for the first-time. The E-cadherin level was not rescued upon genetic loss of LASP1, indicating several layers of repression for E-cadherin in TNBC cells. However, when LASP1 was knocked out in HEK-293 cells, the level of E-cadherin was indeed restored. Similarly, when LASP1 was stably silenced, E-cadherin level was rescued back in MCF7 and MDA-MB-361 luminal breast cancer cell lines in our previous studies [14]. Earlier work also discussed nuclear LASP1 as part of the transcription factor AP-1 [66].

Overall, our study indicates that the CXCR4-LASP1 axis can contribute to the nuclear stability of Snail1 through several mechanisms. This includes the activation of Akt, induction of nuclear localization and/or retention of A20 and LSD1, and a potential for nuclear LASP1 to physically shield and protect Snail1 from GSK-3β induced phosphorylation and subsequent degradation in the cytosol. The resulting nuclear retention of Snail1 may contribute to a migratory and invasive phenotype, which then aids in the many steps of TNBC metastasis in vivo. 

## 4. Materials and Methods 

### 4.1. Cell Culture

The human breast cancer cell lines BT20, MCF7, T47D, BT549, MDA-MB-436, MDA-MB-468, SUM-159, MDA-Bone-Un (MDA-MB-231 cells re-isolated from mouse bone metastatic lesions) and MDA-MB-231 cells were originally obtained from American Type Culture Collection (ATCC, Manassas, VA, USA). The CXCR4 MCF7-series was engineered and characterized previously [52]. The cell lines were cultured in Dulbecco’s modified Eagle’s medium (DMEM), with a supplement of 4 mM L-glutamine, sodium pyruvate, 4.5 g/L D-glucose (GE Healthcare Life Sciences, Pittsburgh, PA, USA; Cat. No. SH30243.01), along with 10% heat-inactivated fetal bovine serum (FBS) (Denville Scientific, Swedesboro, NJ, USA; Cat. No. FB5001-H), penicillin (100 I.U.) and streptomycin (100 µg/mL) (Corning, Corning, NY, USA; Cat. No. 30-002-CI). The cells were maintained in a 5% CO_2_ and 95% humidified air incubator, at 37 °C. 

### 4.2. Generation of LASP1 Knock Out (LASP1-KO) Cells by CRISPR-Cas9 Approach

In order to generate knockout (KO) of LASP1 in MDA-Bone-Un and HEK-293 cells, LASP1 CRISPR/Cas9 knockout (LASP1-KO) plasmids (a set of 3 plasmids) were obtained (Santa Cruz, Dallas, TX, USA; Cat. No. sc-404630). Cells were transfected with 3 µg of plasmid DNA, mixed with 150 µL of the diluted UltraCruz reagent (Santa Cruz; Cat. No. sc-395739), following the instructions from the manufacturer. The transfection media was removed 24 h later and replaced with DMEM complete media. Cells were further maintained for 72 h post-transfection. Subsequently, LASP1-KO cells were sorted for green fluorescent protein (GFP) by flow cytometry and single KO cells isolated by limiting dilution. Non-targeting CRISPR/Cas9 plasmids served as control (Santa Cruz; Cat. No. sc-418922). These plasmids encode the Cas9 nuclease and non-specific 20 nucleotide guide RNAs (denoted CRISPR control). The GFP-sorted cells were cultured, and the level of LASP1 expression in each single cell colony was verified by immunoblotting. HEK-293-HA-CXCR4 LASP1 KO and CRISPR control cells were generated previously [67].

### 4.3. Engineering of LASP1 Mutants

Human LASP1 is phosphorylated on Ser146 and Tyr171 [51,59,60]. These were differentially mutated into either phosphomimetic (S146D and Y171D) or phosphonull (S146A and Y171F) substitutions, by employing PCR based site-directed mutagenesis kit (QuikChange II, Agilent Tech., Santa Clara, CA, USA). The oligonucleotides employed to create the mutations were:

Y171F-forward—CAGTGCCCCGGTTTTCCAGCAGCAAAAG;
Y171F-reverse—CTGGCGCTGCTGGAAAACCGGGGCACTG;
Y171D-forward—CAGTGCCCCGGTTGACCAGCAGCCCCAG;
Y171D-reverse—CTGGGGCTGCTGGTCAACCGGGGCACTG;
S146A-forward—CAGAGCGTCGGGATGCACAGGACGGCAG;
S146A-reverse—CTGCCGTCCTGTGCATCCCGACGCTCTG;
S146D-forward—CCAGAGCGTCGGGATGATCAGGACGGCAGCAGC;
S146D-reverse—GCTGCTGCCGTCCTGATCATCCCGACGCTCTGG

The inserts were ligated into pcDNA3.0 and the mutations were verified by dideoxy sequencing (Eurofins Genomics, Louisville, KY, USA). The mutants were shuttled into pGEX-6P-1 vector subsequently to generate GST-LASP1 WT and the mutant fusion proteins. All final constructs, along with pGEX-6P-1 vector (GST control), were transformed into BL21 bacteria for the production of GST and GST-LASP1 proteins using the previously established standard protocols [68]. 

### 4.4. Preparation and Immunoblotting of Nuclear Extracts 

Intact nuclei and cytosolic fractions were separated by employing the Nuclei EZ Prep kit (Sigma-Aldrich, St. Louis, MO, USA), according to the manufacturer’s instructions. Pure nuclei were extracted from cellular lysates with nuclear extraction buffer (50 mM Tris, pH 8.0, 350 mM NaCl, 1 mM DTT, protease and phosphatase inhibitors (5 mM MgCl_2_ and 50 units of DNAse I), and incubating for 2 h at 4 °C [14]. Total protein in the clarified nuclear lysate was quantified using a Bradford protein assay (Bio-Rad, Hercules, CA, USA; Cat. No. 5000006). Moreover, 25–50 µg of total protein from the nuclear extract was separated by 10% SDS-PAGE and transferred onto nitrocellulose membrane (0.45 μM) overnight. The membranes were then blocked with 5% nonfat dry milk (NFDM) in TBST (20 mM Tris, pH 7.6, 100 mM NaCl, 0.1% Tween-20) for 1 h. Subsequently, the membranes were rinsed with TBST and incubated with primary antibodies in 5% BSA or NFDM for 2 h at 4 °C. Subsequently, the membranes were washed thrice with TBST, and incubated with secondary antibodies (1:5000), conjugated with horseradish peroxidase (HRP) in 1% BSA or NFDM in TBST for 1 h at R.T. The membranes were washed thrice with TBST, and the proteins were detected by enhanced chemiluminescence (ECL) Prime kit (GE Healthcare Life Sciences; Cat. No. RPN2232,). 

### 4.5. Immunoblotting

The 25–50 µg of total or nuclear lysates were separated by 10% SDS-PAGE and transferred onto nitrocellulose membrane (0.45 µm), overnight at 25 °C. Then, the membranes were blocked with 5% nonfat dry milk (NFDM) in TBST (20 mM Tris, pH 7.6, 100 mM NaCl, 0.1% Tween-20). After this, the membranes were washed with TBST and incubated with the following primary antibodies in 5% BSA or NFDM for 2 h at 4 °C: LASP1 8C6 clone (Biolegend, San Diego, CA, Cat. No. 909301), Snail1 (Cell signaling Technology, Danvers, MA, USA, Cat. No. 3879), A20 (Cell signaling Technology, Danvers, MA, USA, Cat. No. 4625), A20 (Santa Cruz Biotechnology, Dallas, TX, USA, Cat. No. 166692), LSD1 (Cell signaling Technology, Danvers, MA, USA, Cat. No. 2139), CXCR4 (BD Bioscience, San Jose, CA, USA, Cat. No. 551852), AKT (Cell signaling Technology, Danvers, MA, USA, Cat. No. 9272), p-AKT S473 (Cell signaling Technology, Danvers, MA, USA, Cat. No. 3527), GSK-3β (Cell signaling Technology, Cat. No. 12456), p- GSK-3β S9 (Cell signaling Technology, Cat. No. 5558), Vimentin (Cell signaling Technology, Cat. No. 5741), E-Cadherin (Cell signaling Technology, Cat. No. 14472), EpCAM (Cell signaling Technology, Cat. No. 2929), FBP1 (Cell signaling Technology, Cat. No. 59172), CK-14 (Thermo Fisher LL002, MA511599, Waltham, MA, USA), CYK 18 (Thermo Fisher LL002), Lamin A/C (Cell signaling Technology, Cat. No. 4777), and β-tubulin (Sigma/Aldrich, St. Louis, MO, Cat. No. T0198). 

Then, the membranes were washed thrice with TBS-T and incubated with Goat anti-Mouse IgG (H+L) Superclonal^TM^ Secondary Ab conjugated to HRP (Thermo Scientific, Rockford, IL, USA, Cat No. A28177) or Goat anti-Rabbit IgG (H+L) Superclonal^TM^ Secondary Ab conjugated to HRP (Thermo Scientific, Cat No. A27036). The membranes were washed thrice with TBS-T and the proteins were detected by ECL Prime kit (GE Healthcare Life Sciences; Cat. No. RPN2232) and HyBlot ES^TM^ Autoradiography Film (Denville Scientific; Cat. No. E3212). Densitometry of the Western blots was performed using ImageJ 1.44 software. Calculation of fold change is given in the figure legends. 

### 4.6. Nuclear co-Immunoprecipitation 

Serum-starved Bone-Un Cells were stimulated with 20 nM CXCL12 (ligand for CXCR4) for 20 min at 37 °C. Some batches of cells were pre-incubated with AMD3465 prior to the addition of CXCL12. The clarified nuclear lysate was prepared as described in aforementioned ‘*Immunoblotting of nuclear extract’*. LASP1 was immunoprecipitated with a mouse monoclonal anti-LASP1 clone-8C6, which was generated against amino acid sequence 151–162 (SYRRPLEQQQPH) within the LASP1 linker domain (not present in LASP2), and associated proteins were analyzed as described [12,14,69].

### 4.7. Glutathione S-Transferase (GST) Pulldown Assay

For the GST pull-down assay, glutathione S-transferase (GST), GST-LASP1, and its domain fusion proteins constructs (GST-LIM, GST-NRLD (Nebulin repeats and linker domain), GST-SH3 (C-terminal domain)) were expressed in BL21 bacteria. The GST fusion proteins were isolated as described [12,14,67]. The beads were aliquoted and stored at −80 °C. 

Furthermore, 1.5 nmol of the GST-LASP1 (Full length), GST-LIM (N-terminal domain), GST-NRLD, GST-SH3 (C-terminal domain) and GST bound to glutathione beads were washed thrice with binding buffer (50 mM Tris-HCl pH 7.5, 0.05% Triton X-100, 100 mM NaCl - Co-IP buffer) and incubated with nuclear lysate overnight at 4 °C. Subsequently, the beads were washed thrice with Co-IP buffer and the bound proteins were eluted with 2×Laemmli buffer and resolved by 10% sodium dodecyl sulfate-polyacrylamide gel electrophoresis (SDS-PAGE). This was followed by immunoblotting for proteins associated to LASP1 and its various domains. 

### 4.8. Subcellular Localization of Snail1

The CRISPR control and LASP1-KO cells were seeded onto glass cover slips coated with Collagen IV, and allowed to attach and spread overnight. The cells were fixed on the following day with 4% paraformaldehyde and permeabilized by Triton X-100. After overnight incubation with Snail1 antibody (Cell Signaling Technology, Cat. No. 3879), the coverslips were washed thrice with phosphate-buffered saline, pH 7.5 (PBS). Snail1 was visualized with the Alexa Fluor 488 conjugated goat anti-rabbit secondary antibody at RT for 2 h. Cellular outline was visualized by decorating the filamentous actin (F-actin) with FITC-Phalloidin (Life Technologies, Carlsbad, CA, USA, Cat. No. A12379) for 30 min, followed by DRAQ5 (Thermo Scientific, Cat No. 62251) for 10 min, to visualize the nuclei at RT. The coverslips were air-dried and mounted on glass slides. The subcellular distribution of Snail1 was analyzed by Leica TCS SP5 multiphoton laser scanning confocal microscopy. Analysis of images was performed using the Leica Application Suite X software. 

### 4.9. Chromatin Immunoprecipitation (ChIP) Assay

The chromatin was prepared as described [70]. Briefly, serum starved ‘Bone-Un’ cells were stimulated with and without 20 nM CXCL12, and the protein-DNA complexes were cross-linked with 37% formaldehyde, diluted to a final concentration of 1% for 10 min. The formaldehyde was quenched with 2.5 M glycine diluted to a final concentration of 125 mM for 10 min at RT. Cells were washed and scraped into PBS, pH 7.5, pelleted, washed again and treated with ‘Lysis buffer I’ (50 mM HEPES-KOH, pH 7.5, 140 mM NaCl, 1 mM EDTA, 10% glycerol, 0.5% IGEPAL CA-630, 0.25% Triton X-100, 1× protease inhibitors), followed by nutation for 10 min at 4 °C to release the nuclei. The nuclei were recovered by centrifugation and resuspended in 1 mL ‘Lysis buffer II’ (10 mM Tris-HCl, pH 8.0, 200 mM NaCl, 1 mM EDTA, and 0.5 mM EGTA, 1× protease inhibitors), and nutated for 10 min at 4 °C. After centrifugation, the pellet was resuspended in ‘Lysis buffer III’ (10 mM Tris-HCl, pH 8.0, 100 mM NaCl, 1 mM EDTA, 0.5 mM EGTA, 0.1% sodium deoxycholate, 0.5% N-lauroylsarcosine and 1× protease inhibitors). Samples were then placed on ice and sonicated in 30 s pulses set at 50% amplitude, for a total of 8 min. After the sonication, the samples were centrifuged at 13,000× *g* for 10 min at 4 °C, and the purified chromatin extract (supernatant) was collected. The chromatin concentration was quantified by Nanodrop. Next, 100 μg of chromatin extract was pre-cleared and incubated with isotype control IgG or anti-LASP1 antibody overnight at 4 °C. On the next day, 80 μL of ‘Protein A/G plus Fast Flow’-agarose beads (GE Healthcare Life Sciences) were added, and the incubation continued for an additional 4 h. The beads were washed thrice with wash buffer (50 mM HEPES-KOH, pH 7.5, 500 mM LiCl, 1 mM EDTA, 1.0% IGEPAL CA-630, 0.7% sodium deoxycholate), and finally eluted in elution buffer (50 mM Tris-HCl, pH 8.0, 10 mM EDTA, 1.0% SDS). The cross-links were reversed by heating at 65 °C overnight (~15 h), and the chromatin was extracted with phenol-chloroform, and the DNA was precipitated with 80% ethanol from the aqueous phase. The precipitated DNA was washed twice with 70% ethanol, and the air-dried pellet was resuspended in diluted TE (1 mM Tris, pH 8.0, 0.5 mM EDTA). Furthermore, 3 µL of the final resuspended DNA was subjected to qPCR by employing the human E-cadherin promoter [(+) 5′-GGAGGGGTCCGCGCTGCTGA and (−)5′-AGCTCACAGGTGCTTTGCAG)] and β-tubulin [(+) 5′ CTGGACCGCATCTCTGTGTACTAC3′) and (−) 5′ GACCTGAGCGAACAGAGTCCAT)] primers. 

### 4.10. Matrigel Invasion Assay

Invasion of CRISPR-control and LASP1-KO cells was assessed by employing a transwell Matrigel assay kit (8-µM pore size) (Corning). Then, 1 × 10^5^ cells/well resuspended in 200 µL serum-free medium were added to the upper Matrigel pre-coated chamber (BD Bioscience). The lower chamber of the transwell plate was filled with 600 μL of serum free DMEM, with 20 nM CXCL12 as the chemoattractant. After incubation overnight at 37 °C, the cells attached on the inner surface of the lower chamber were fixed with 100% methanol. The fixed cells were visualized by staining with 0.1% crystal violet (Sigma-Aldrich). The cells that invaded through Matrigel were counted. All the experiments were performed in triplicate. 

### 4.11. Isolation of ALDH-Positive BCSCs by FACS

ALDH-positive BCSCs were isolated from MDA-Bone-Un LASP1 CRISPR control (CC) and LASP1-knockout cells (KO), based on ALDH activity, by employing the ALDEFLUOR™ assay kit (Stem Cell Technologies, Vancouver, BC, Canada, Cat. #−01700) using a method described earlier [71]. 

### 4.12. Kaplan–Meier Survival Analysis

The probability of relapse-free survival (RFS) of basal-like breast cancer patients with a high vs. low expression of LASP1 and SNAI1 was analyzed using the Kaplan–Meier survival analysis (https://kmplot.com/). The 10-year RFS was assessed in 112 TNBC patients (Grade 3 tumors) with the hazard ratio (HR) and log rank *p* value.

### 4.13. Statistical Analysis 

Analysis of the data was performed by employing the software GraphPad Prism ver. 7.0. Statistical significance between groups was determined by Student’s t-test. All the experiments had three biological replicates.

## 5. Conclusions

We determined that the CXCR4-LASP1 axis contributes to the stability of the EMT-TF Snail1, which is a key mediator of TNBC metastasis. 

## Supplementary Materials

The following are available online at https://www.mdpi.com/2072-6694/12/9/2372/s1, Figure S1: Genetic loss of LASP1 alters EMT signaling protein in TNBC cells.
