# Supplementary material for: Role of the CXCR4-LASP1 Axis in the Stabilization of Snail1 in Triple-Negative Breast Cancer
Source: Cancers (Basel). 2020 Aug 21;12(9):2372. doi: 10.3390/cancers12092372 (PMC7563118; doi:10.3390/cancers12092372)

Figure 1B

Exposure 1

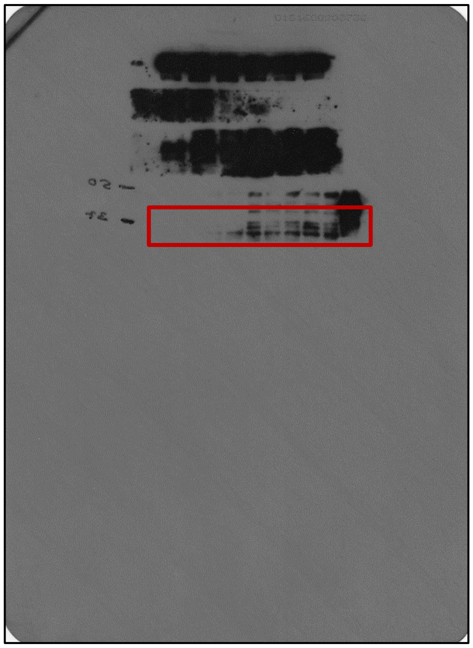

CXCR-4-46 kDa

Exposure 2

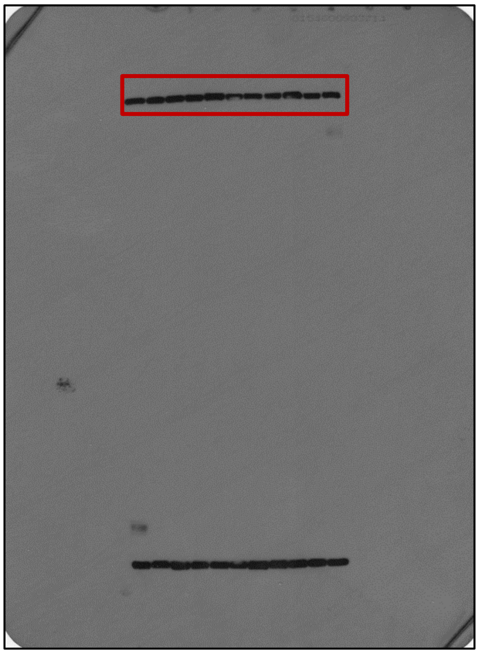

β-tubulin-55 kDa

Figure 1C

Exposure 1

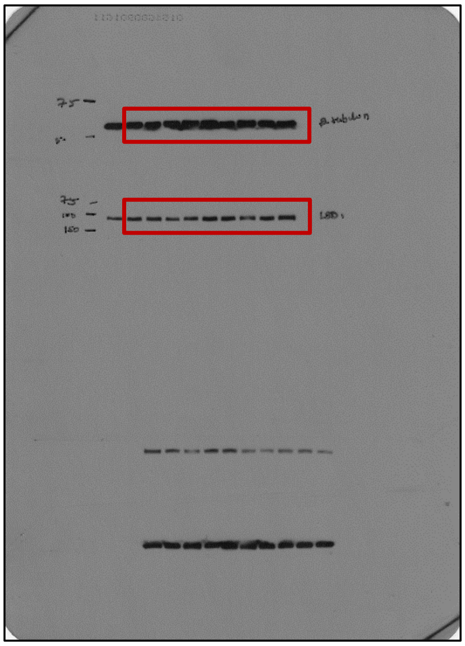

β-tubulin -55 kDa
LSD1-110 kDa

Exposure 2

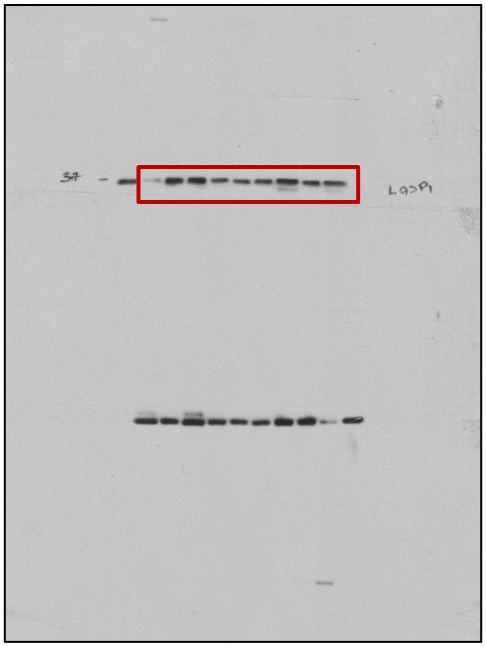

LASP1-37 kDa

Exposure 3

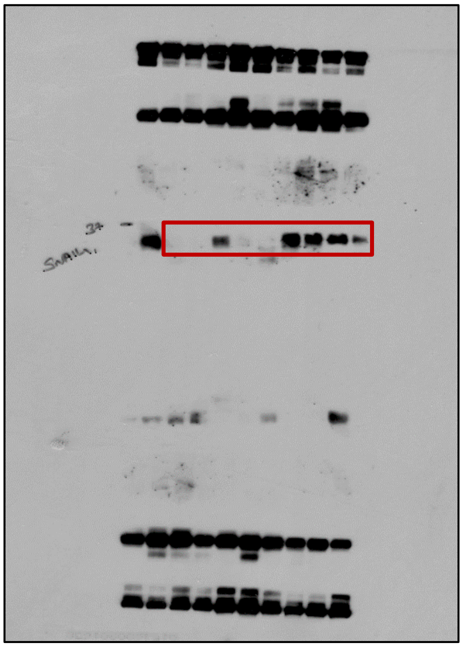

Snail1-29 kDa

Exposure 4

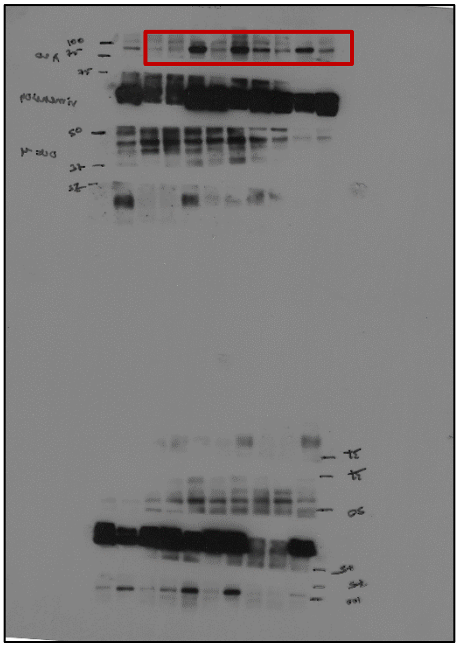

A20-90 kDa

Exposure 5

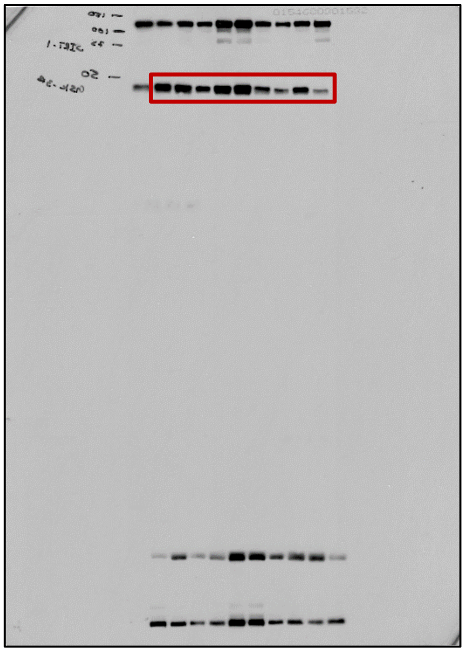

GSK-3β-46 kDa

Figure 2A

Exposure 1

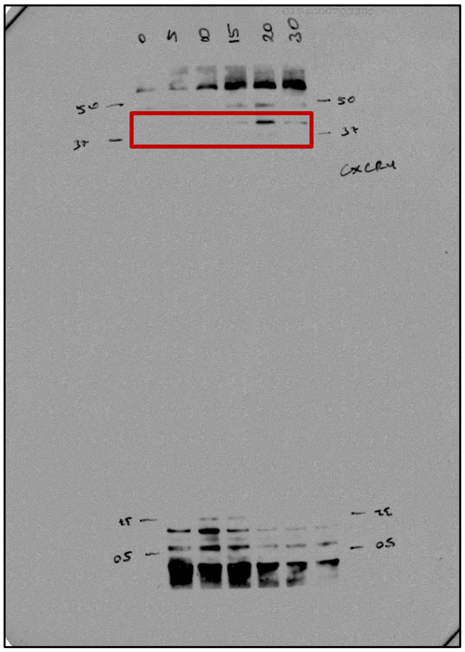

CXCR4-46 kDa

Exposure 2

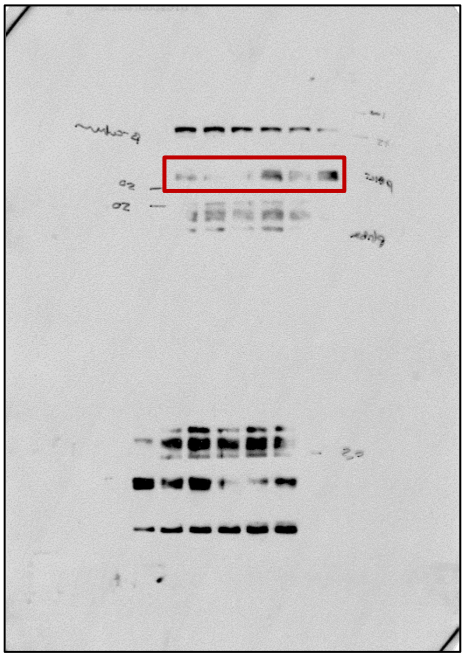

pS473-Akt-60 kDa

Exposure 3

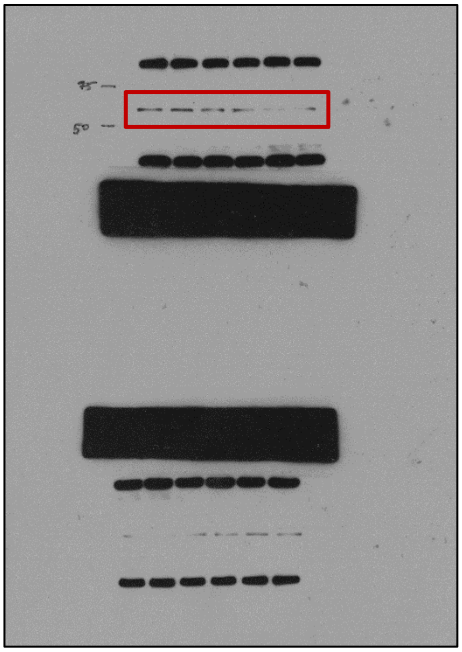

Akt-60 kDa

Exposure 4

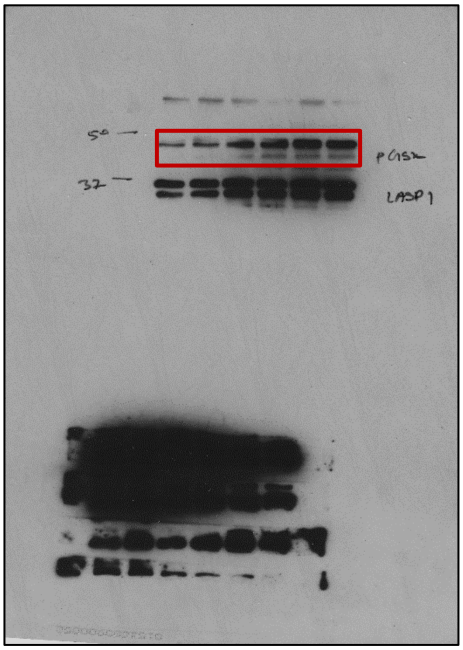

pS9-GSK-3β- 46 kDa

Exposure 5

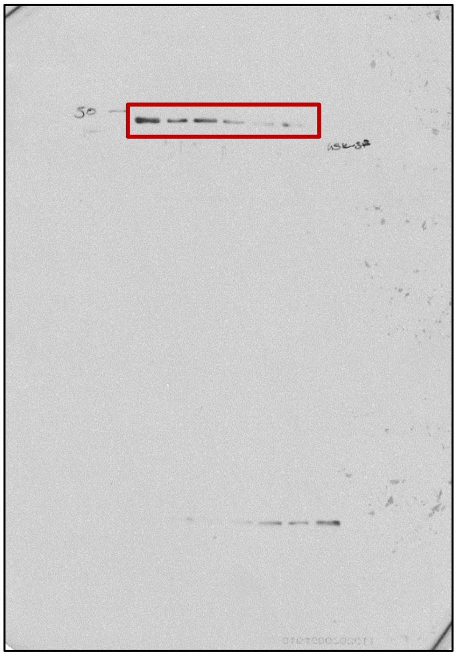

GSK-3β- 46 kDa

Exposure 6

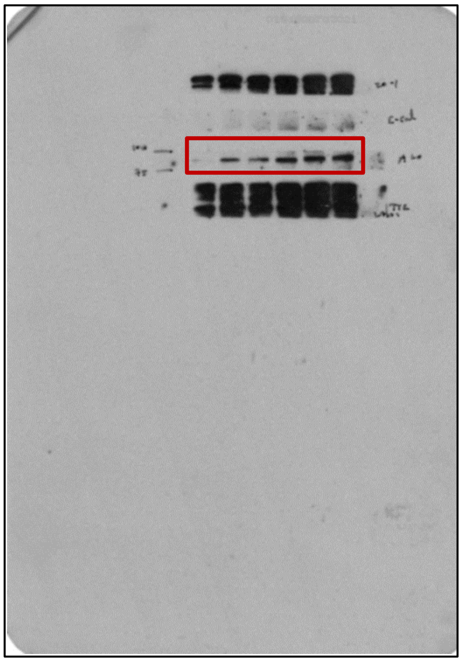

A20-90 kDa

Exposure 7

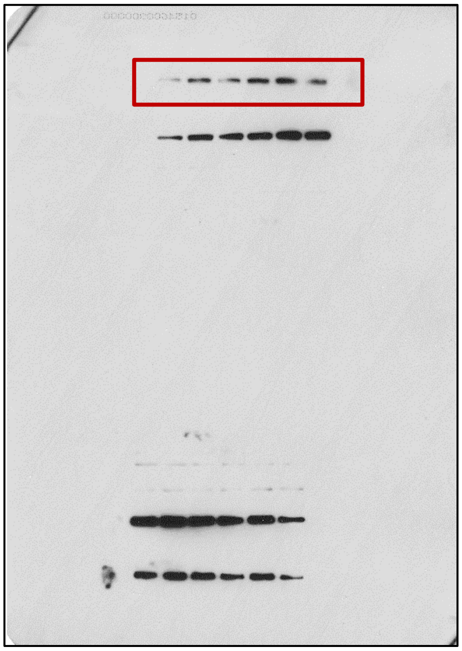

LSD1-110 kDa

Exposure 8

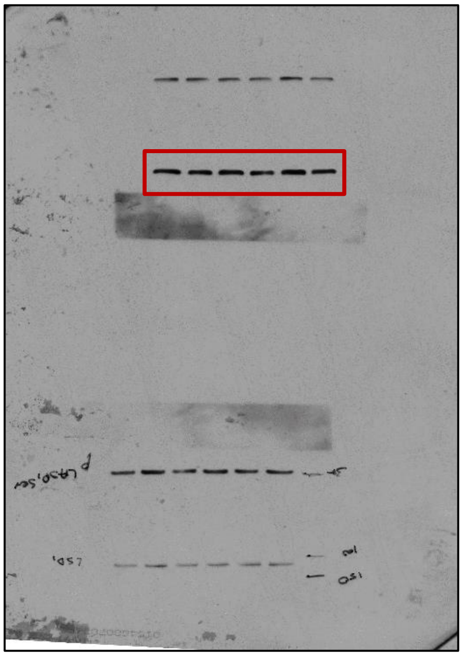

pS146-LASP1

Exposure 9

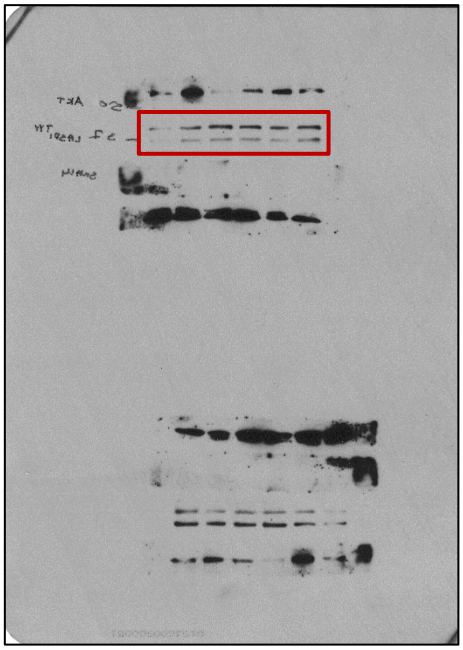

pY171-LASP1

Exposure 10

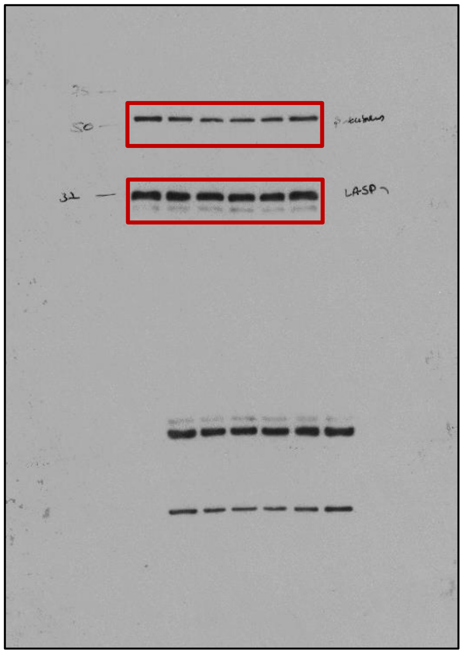

β-tubulin – 55 kDa
LASP1- 37 kDa

Exposure 11

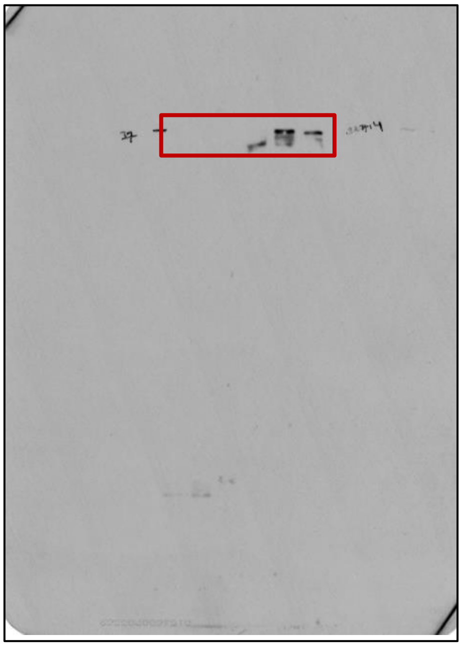

Snail1-29kDa

Exposure 12

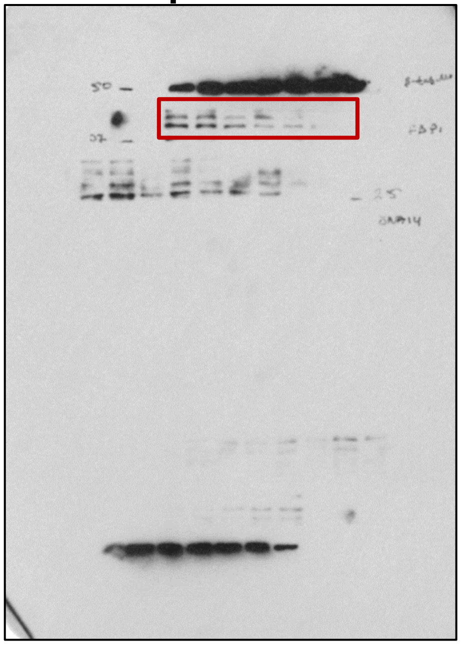

FBP1-39kDa

Exposure 13

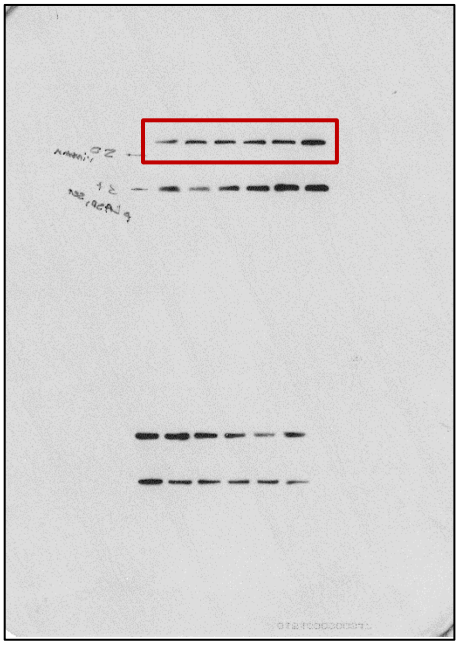

Vimentin-57 kDa

Figure 2B

Exposure 1

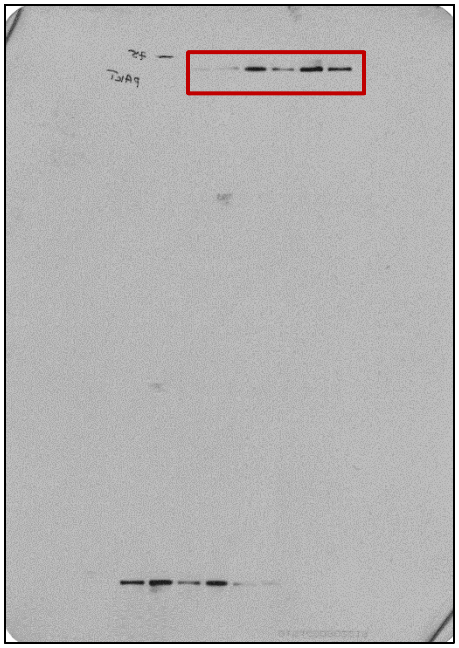

pS473-Akt-60 kDa

Exposure 2

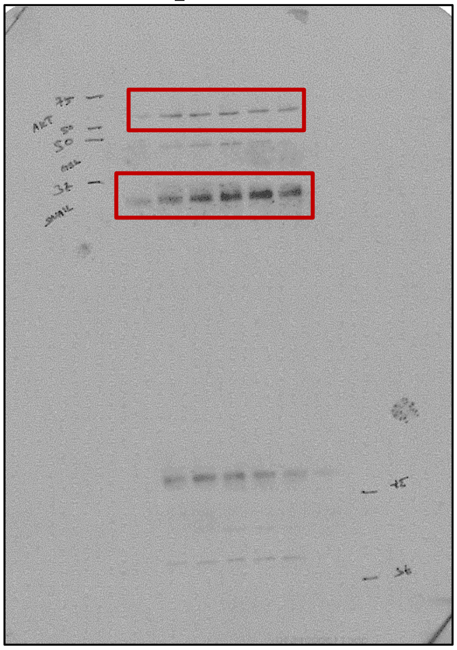

Akt-60 kDa
Snail1-29kDa

Exposure 3

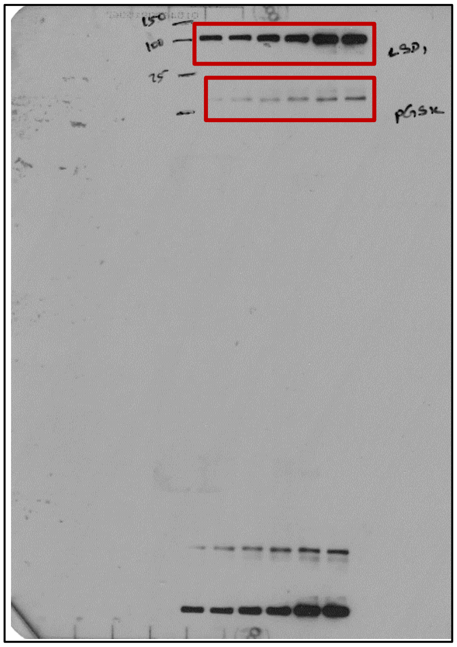

LSD1-110 kDa,
pS9-GSK-3β- 46 kDa

Exposure 4

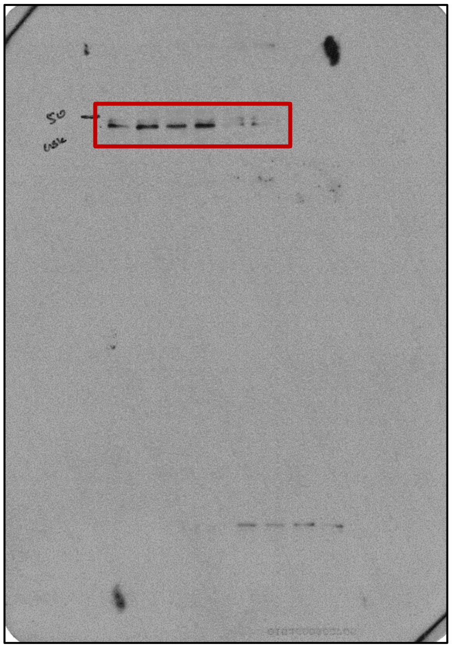

GSK-3β- 46 kDa

Exposure 5

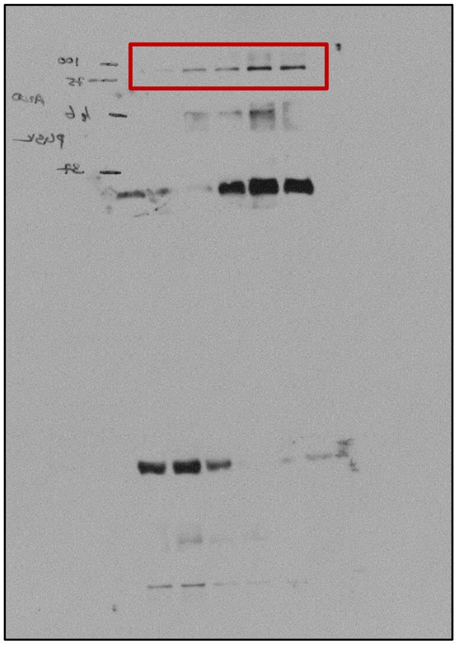

A20-90 kDa

Exposure 6

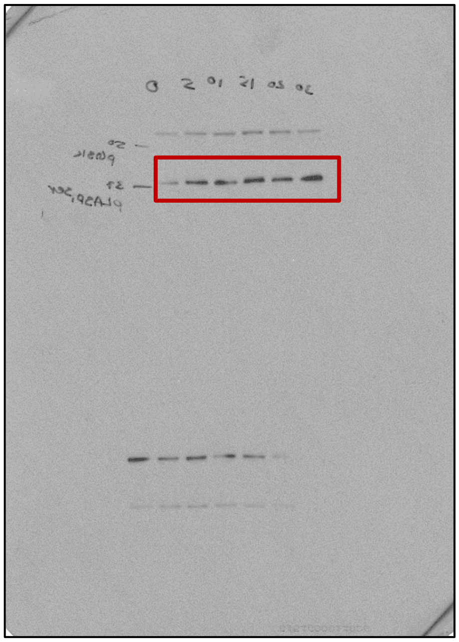

pS146-LASP1

Exposure 7

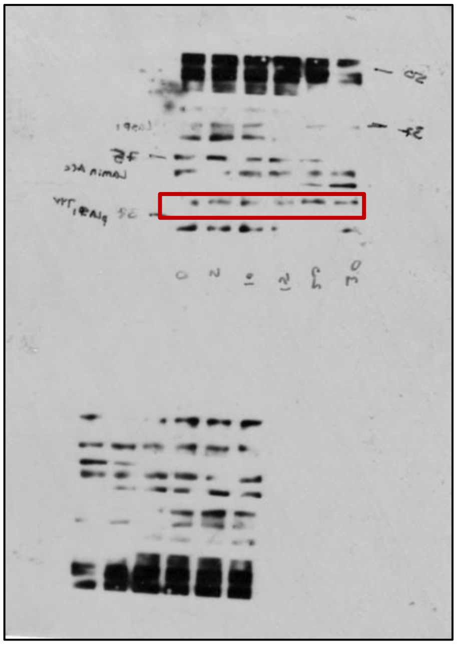

pY171-LASP1

Exposure 8

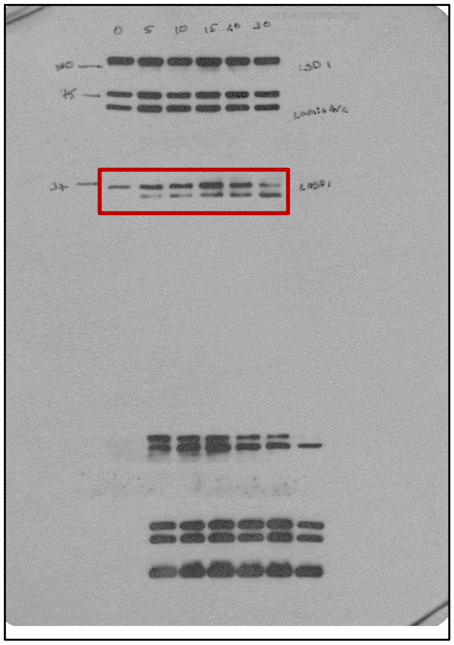

LASP1- 37 kDa

Exposure 9

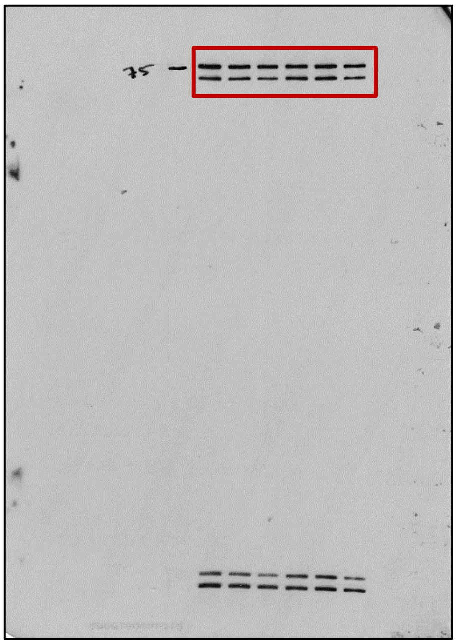

Lamin A/C- 72 kDa

Figure 2C

Exposure 1

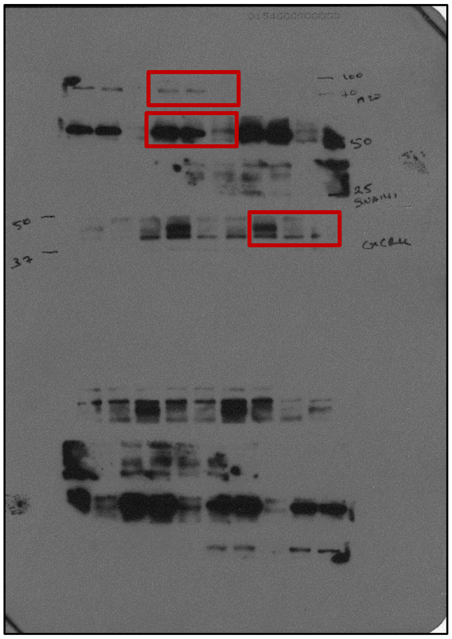

A20-90 kDa, Vimentin- 55 kDa
CXCR-46 kDa

Exposure 2

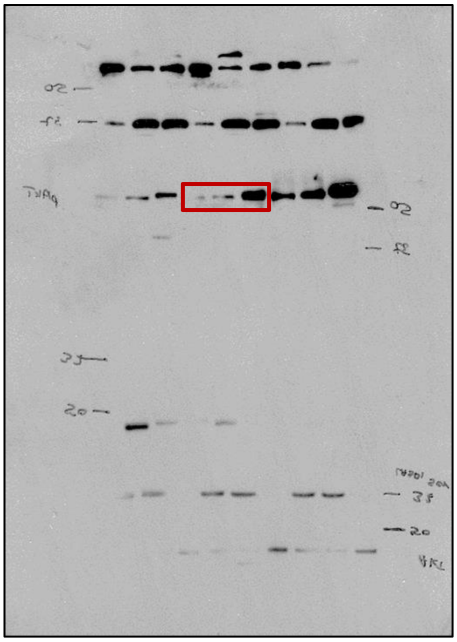

pS473-Akt-60 kDa

Exposure 3

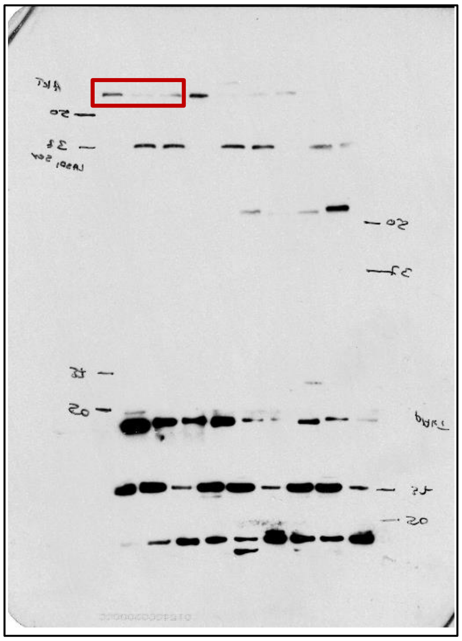

Akt-60 kDa

Exposure 4

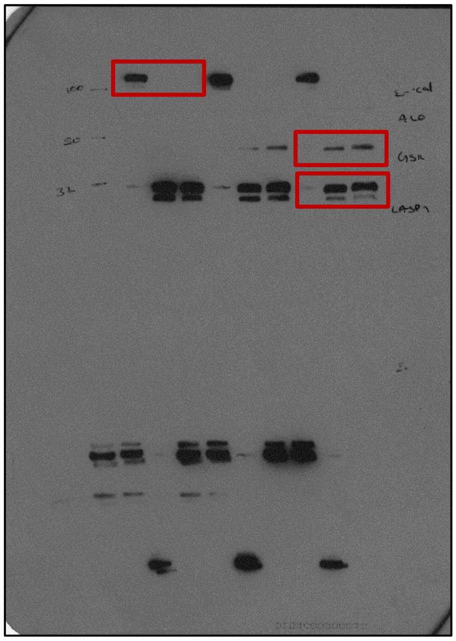

E-cadherin-120 kDa, GSK-3β- 46kDa
LASP1-37 kDa

Exposure 5

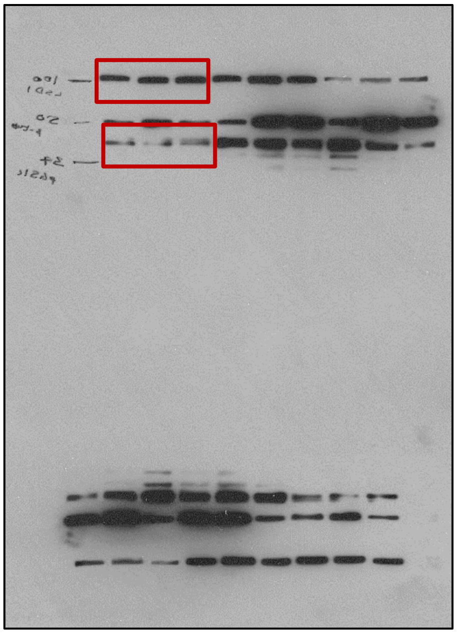

LSD1-110 kDa
pS9-GSK-3β- 46kDa

Exposure 6

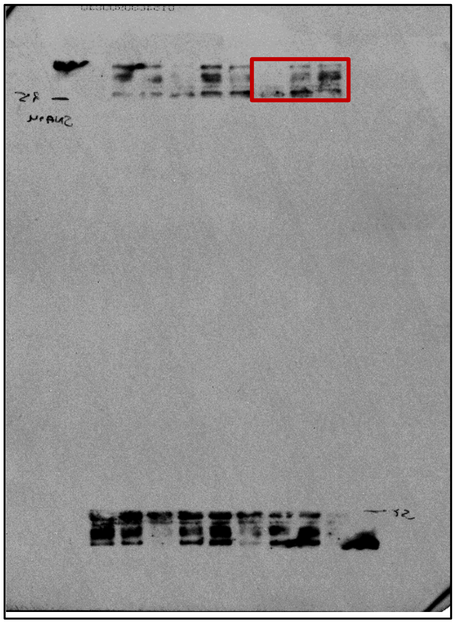

Snail1-29kDa

Exposure 7

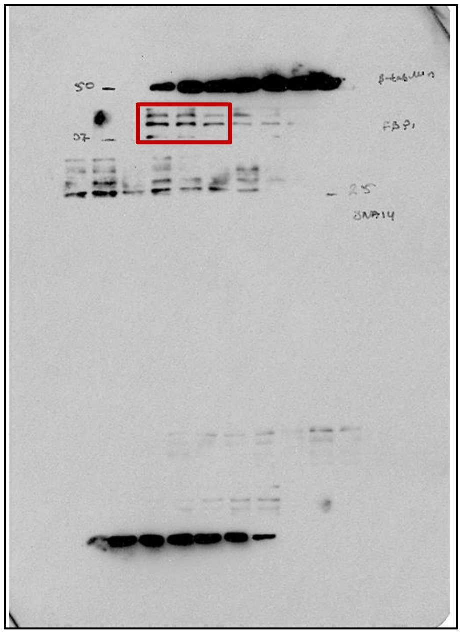

FBP1-39 kDa

Exposure 8

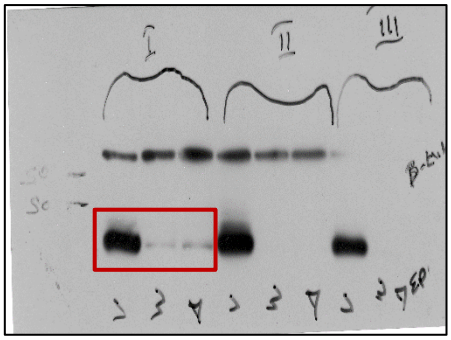

EpCAM--40 kDa

Exposure 9

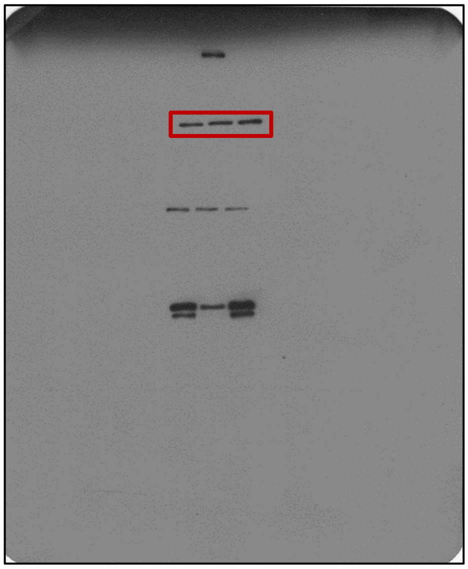

β-tubulin-55 kDa

Figure 3B

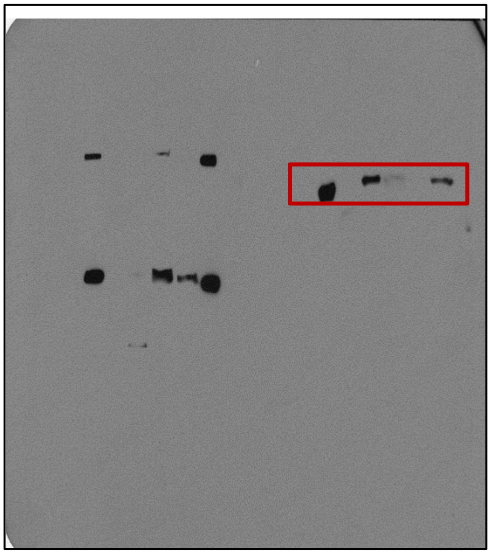

Snail1-29 kDa

Figure 3C

Exposure 1

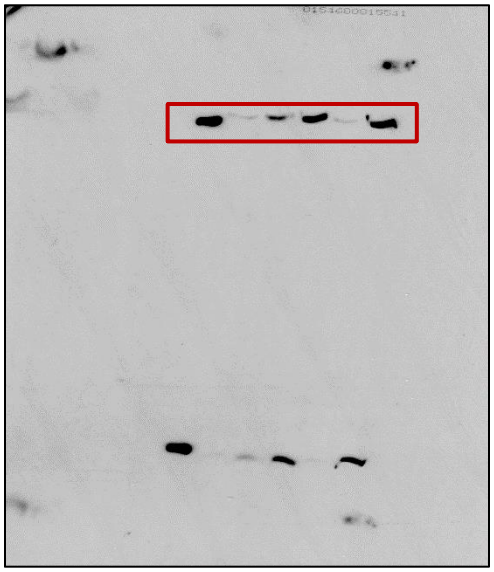

A20-90 kDa

Exposure 2

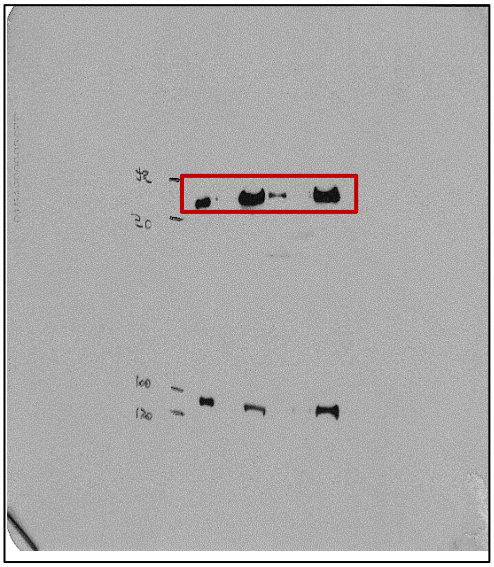

LSD1-110 kDa

Figure 3D

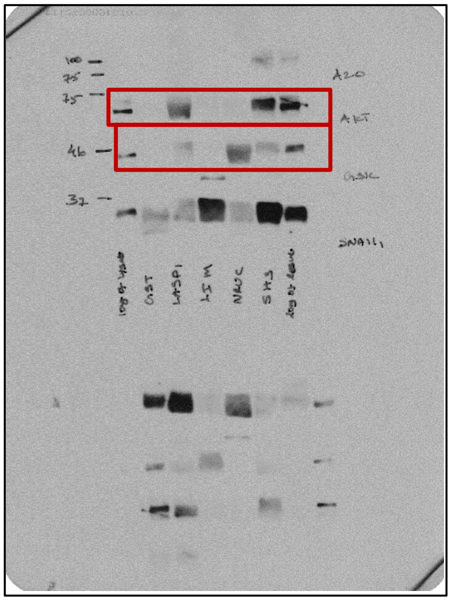

Akt-60 kDa
GSK-3β- 46 kDa

Figure 4A

Exposure 1

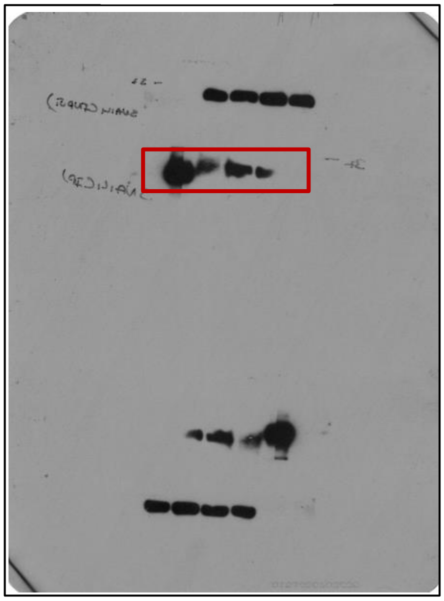

Snail1-29 kDa

Exposure 2

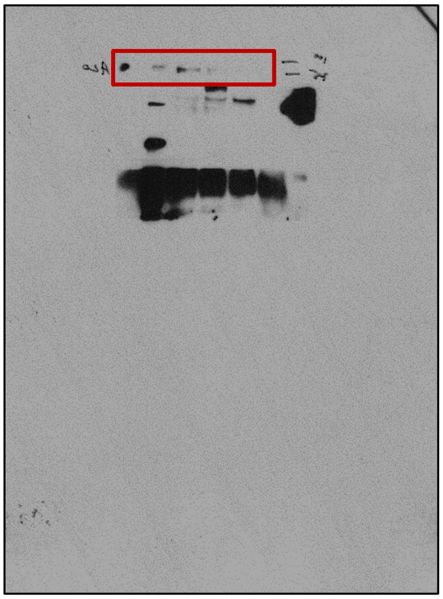

A20-90 kDa

Exposure 3

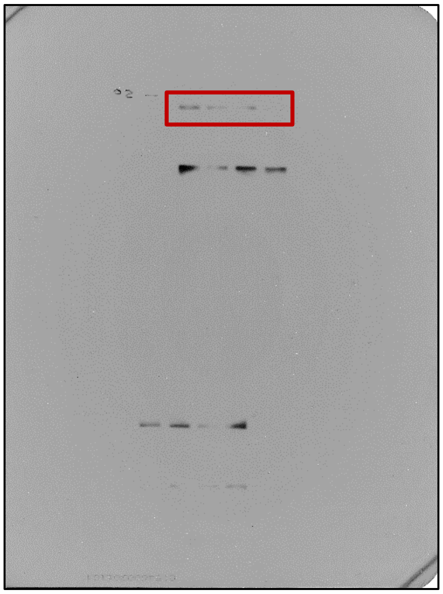

GSK-3β- 46 kDa

Exposure 4

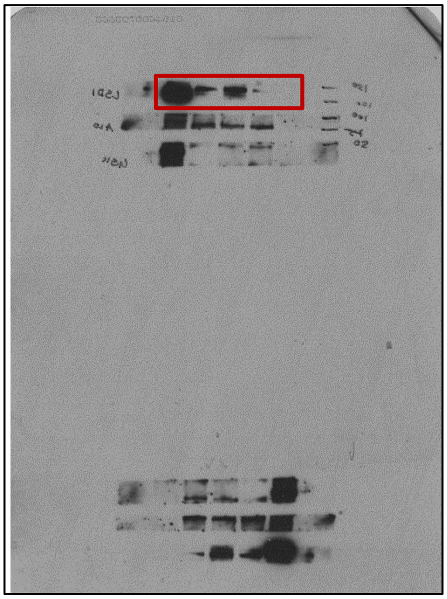

LSD1-110 kDa

Exposure 5

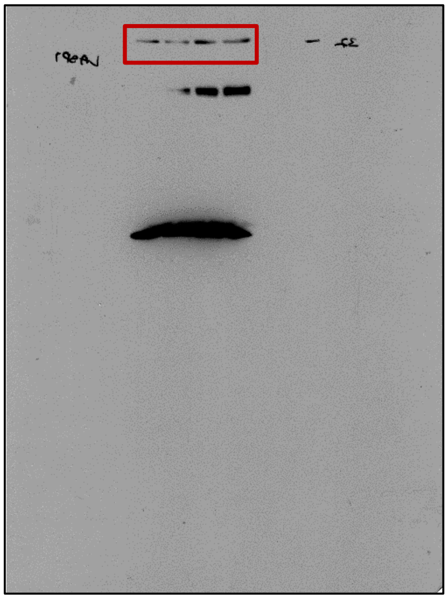

LASP1-37 kDa

Input Lysates

Exposure 1

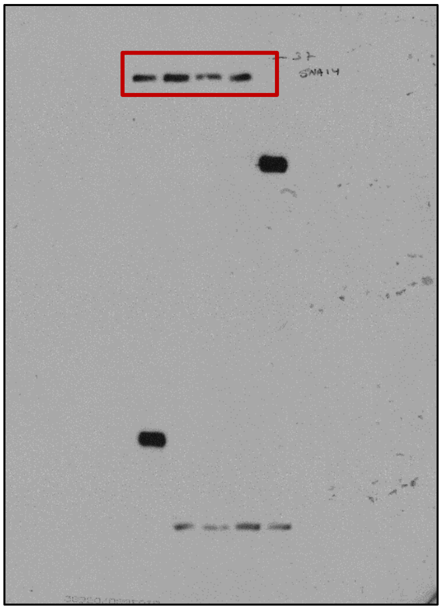

Snail1-29 kDa

Exposure 2

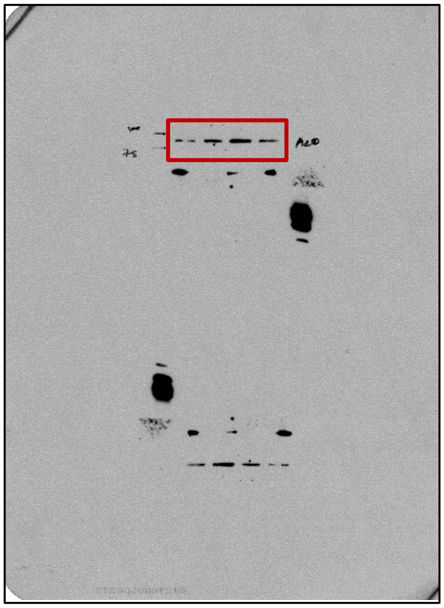

A20-90 kDa

Exposure 3

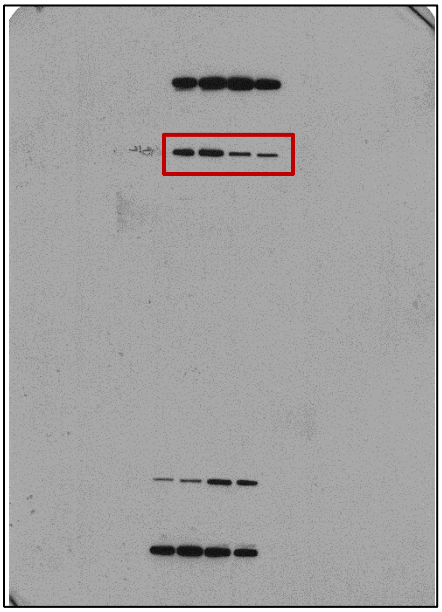

GSK-3β- 46 kDa

Exposure 4

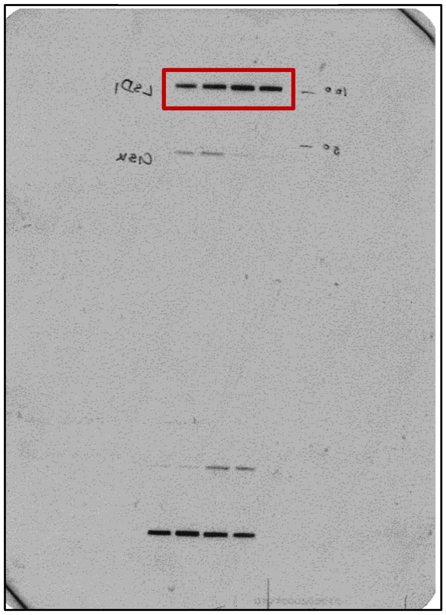

LSD1-110 kDa

Exposure 5

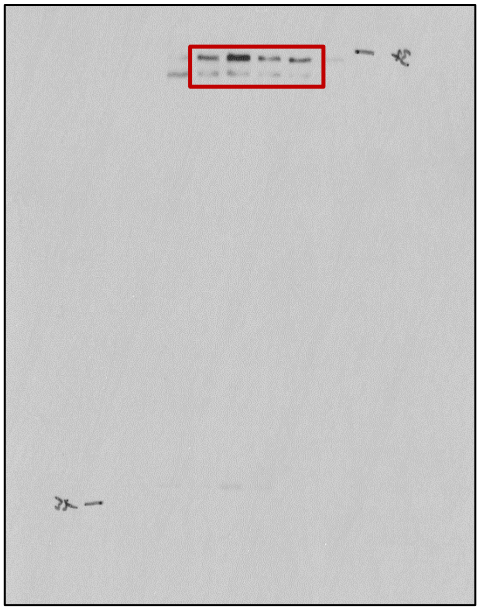

LASP1-37 kDa

Figure 4B

Exposure 1

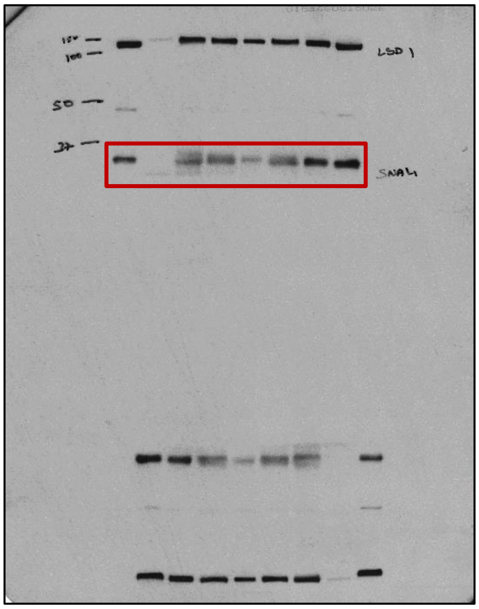

Snail1-29 kDa

Exposure 2

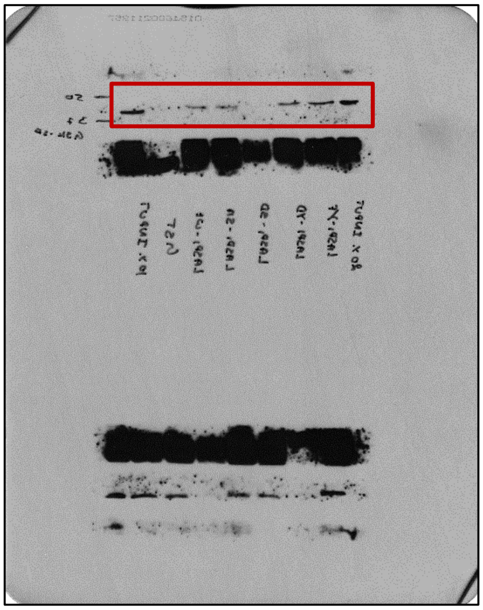

GSK-3β- 46 kDa

Exposure 3

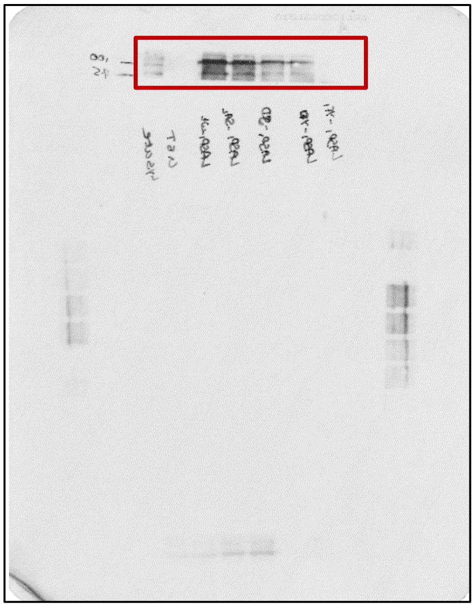

A20-90 kDa

Exposure 4

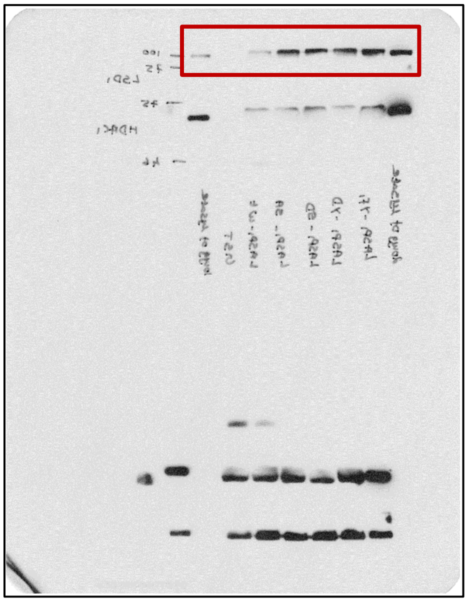

LSD1-110 kDa

Figure 5A

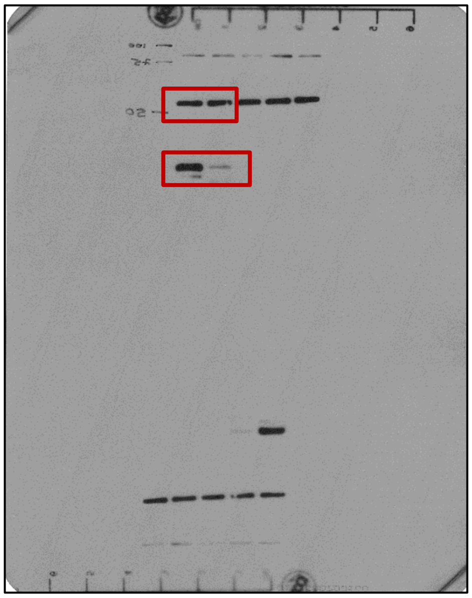

β-tubulin -55 kDa
LASP1-37 kDa

Figure 5C

Exposure 1

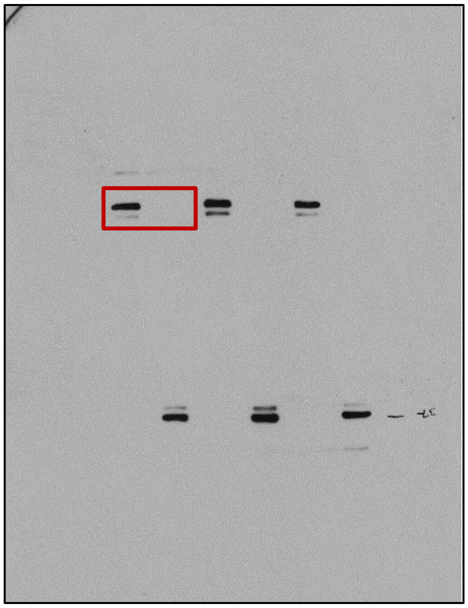

LASP1-37 kDa

Exposure 2

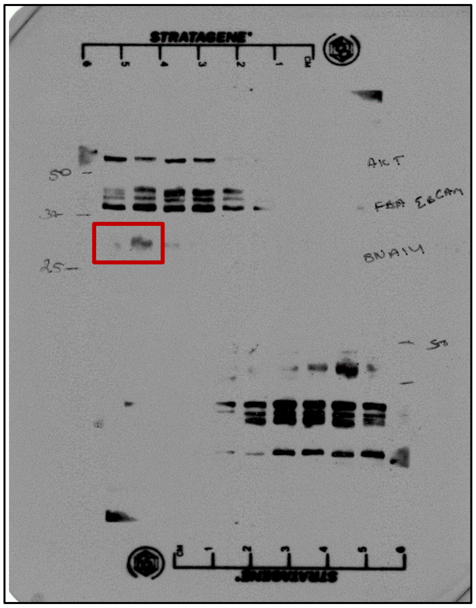

Snail1-29 kDa

Exposure 3

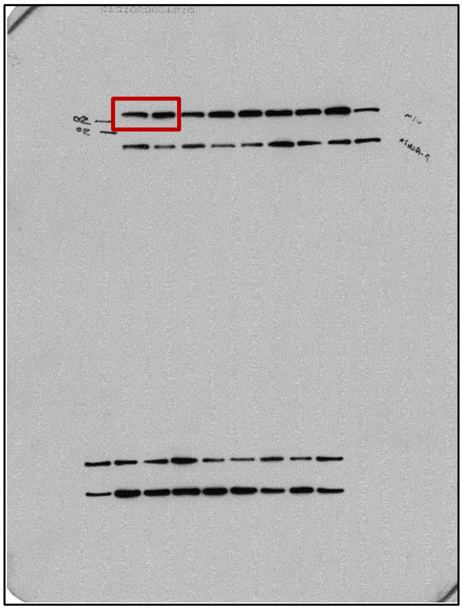

Vimentin-57 kDa

Exposure 4

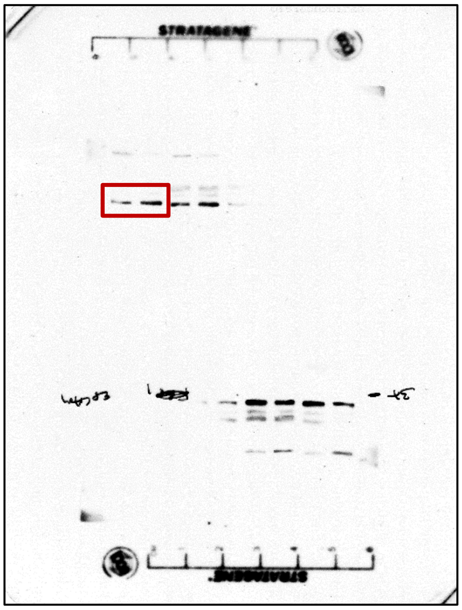

EpCAM-40 kDa

Exposure 5

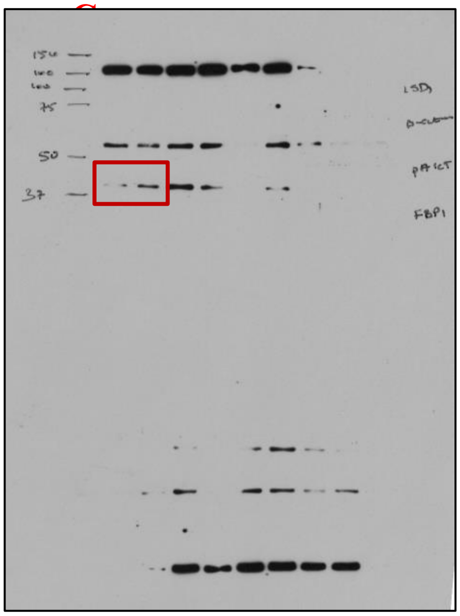

FBP1-39 kDa

Exposure 6

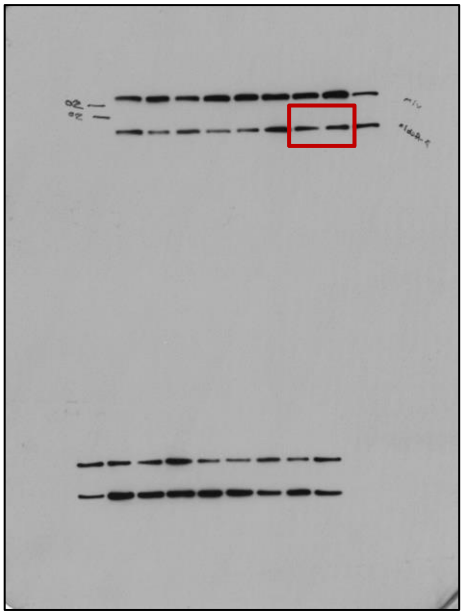

β-tubulin-55 kDa

Exposure 7

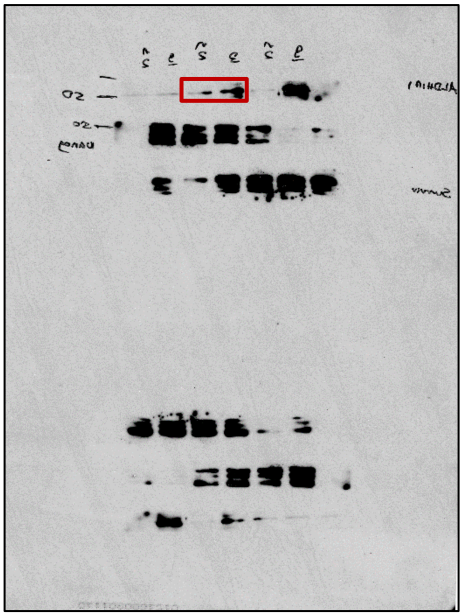

ALDH1A1-55 kDa

Figure 5D

Exposure 1

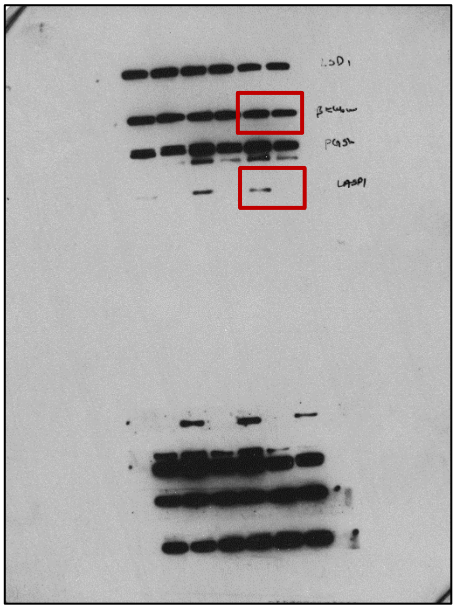

β-tubulin-55 kDa
LASP1-39 kDa

Exposure 2

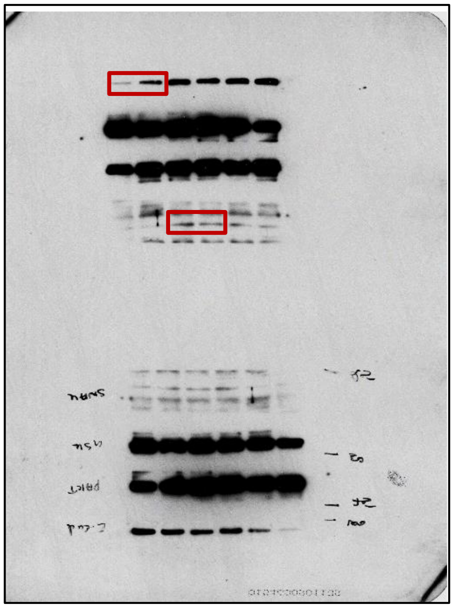

E-cadherin-120 kDa
Snail1-29 kDa

Exposure 3

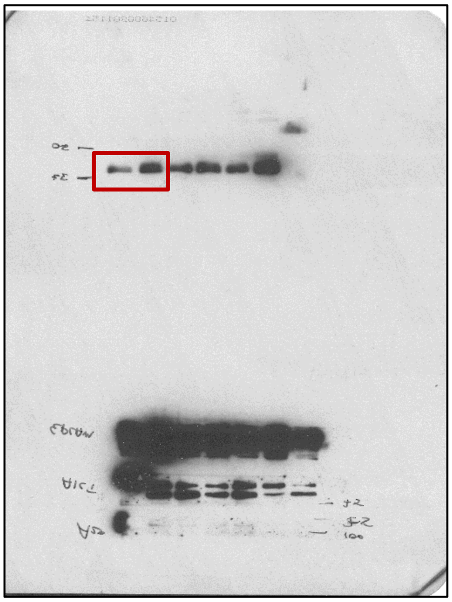

EpCAM-40 kDa

Exposure 4

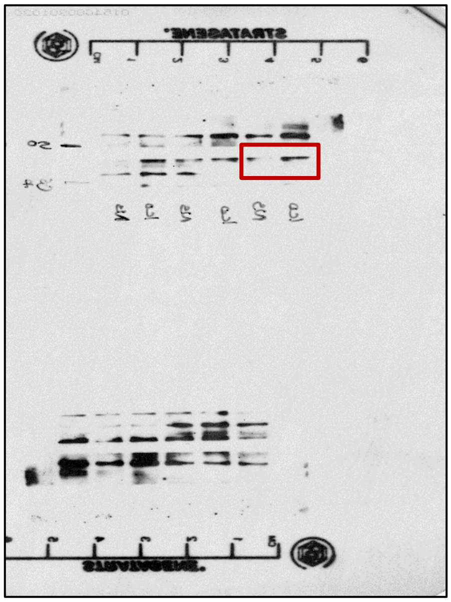

FBP1-39 kDa

Exposure 5

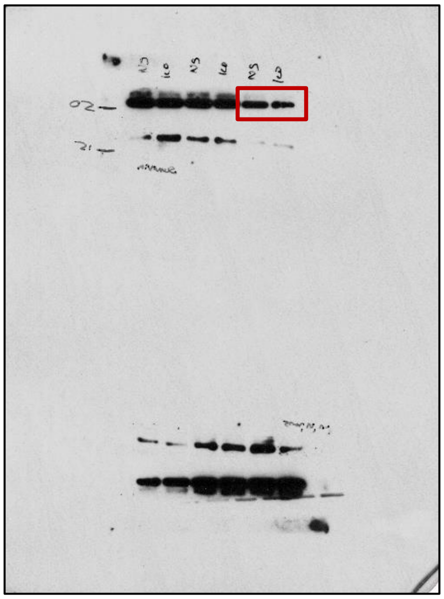

Vimentin-57 kDa

Exposure 6

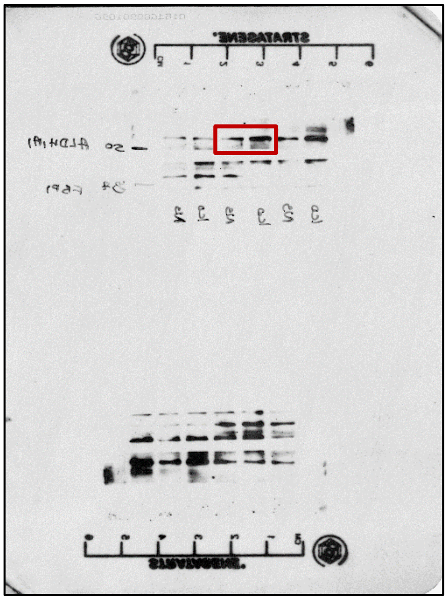

ALDH1A1 – 55 kDa

Figure 5F

Exposure 1

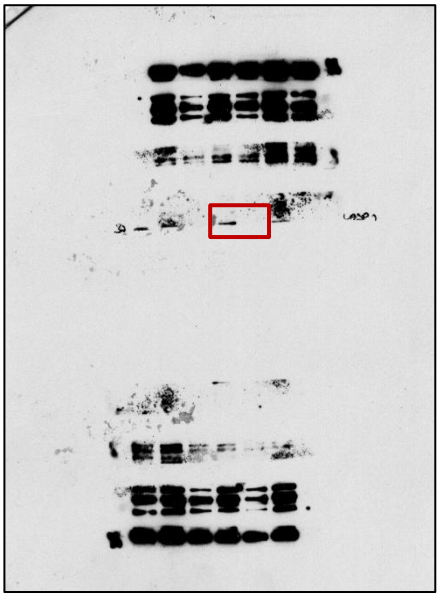

LASP1-37 kDa

Exposure 2

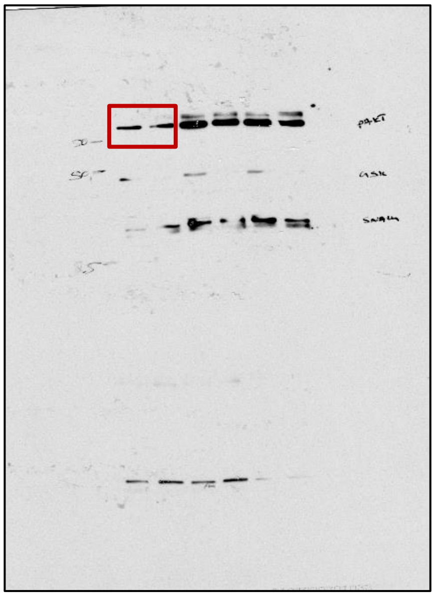

pS473-Akt-60 kDa

Exposure 3

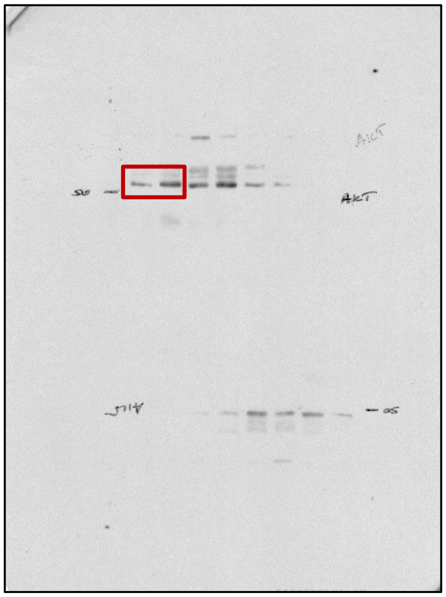

Akt-60 kDa

Exposure 4

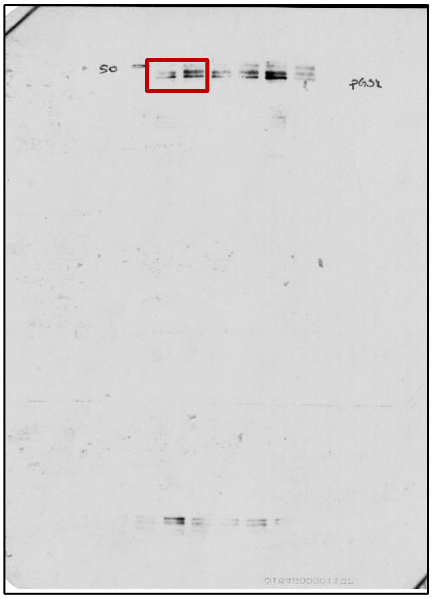

pS9-GSK-3β-46 kDa

Exposure 5

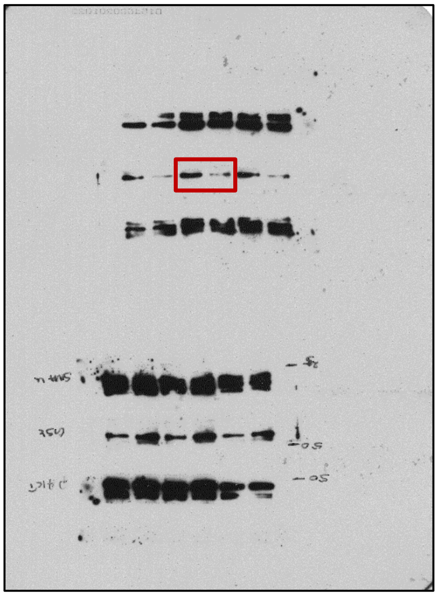

GSK-3β-46 kDa

Exposure 6

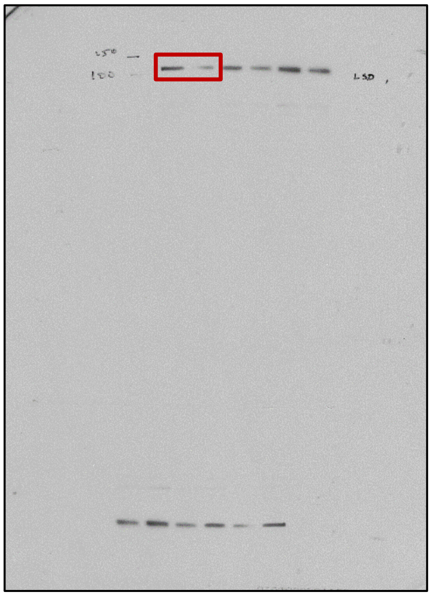

LSD1-110 kDa

Exposure 7

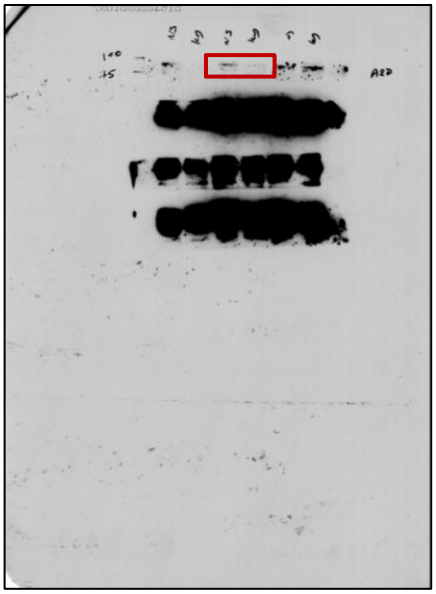

A20-90 kDa

Exposure 8

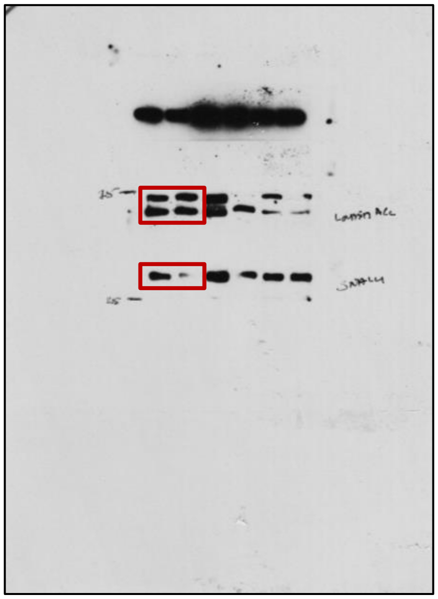

Lamin A/C-72 kDa
Snail1-90 kDa

Figure S1

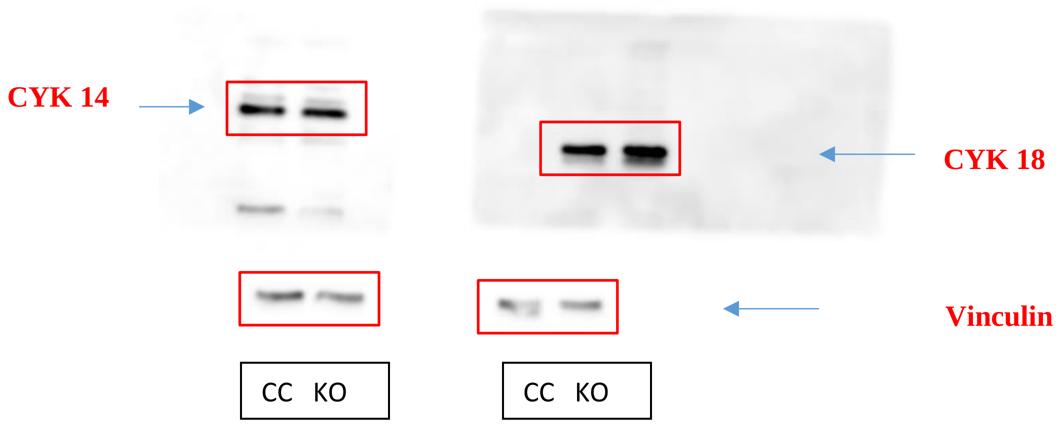

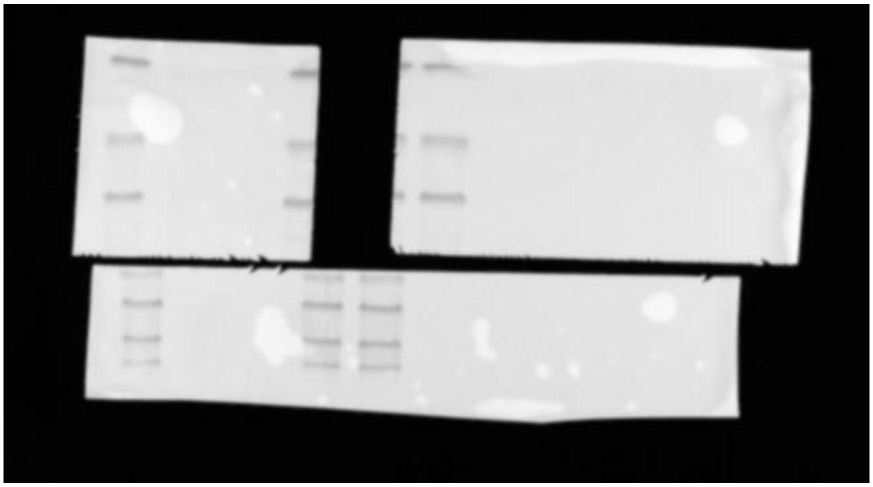

Supplement: Supplementary file 1 [file cancers-12-02372-s001.pdf]
